# Genetic diversity and population dynamic of *Ziziphus jujuba* var. *spinosa* (Bunge) Hu ex H. F. Chow in Central China

**DOI:** 10.1002/ece3.9101

**Published:** 2022-07-24

**Authors:** Shuhui Du, Xiaoyan Hu, Xiuyun Yang, Wendong Yu, Zhaoshan Wang

**Affiliations:** ^1^ College of Forestry, Shanxi Key Laboratory of Cultivation and Development on Functional Oil Trees in the Northern China Shanxi Agricultural University Taigu Jinzhong China; ^2^ College of Horticulture and Plant Protection Yangzhou University Yangzhou China; ^3^ State Key Laboratory of Tree Genetics and Breeding, Key Laboratory of Silviculture of the State Forestry Administration, Research Institute of Forestry Chinese Academy of Forestry Beijing China

**Keywords:** chloroplast genome, genetic structure, nucleotide variation, population dynamic, single‐copy nuclear gene markers, *Ziziphus jujuba* var. *spinosa*

## Abstract

Phylogeographic research concerning Central China has been rarely conducted. Population genetic and phylogeography of *Ziziphus jujuba* var. *spinosa* (also called sour jujube) were investigated to improve our understanding of plant phylogeographic patterns in Central China. Single‐copy nuclear gene markers and complete chloroplast genome data were applied to 328 individuals collected from 21 natural populations of sour jujube in China. Nucleotide variation of sour jujube was relatively high (*π* = 0.00720, *θ*_w_ = 0.00925), which resulted from the mating system and complex population dynamics. Analysis of molecular variation analysis revealed that most of the total variation was attributed to variation within populations, and a high level of genetic differentiation among populations was detected (*F*_st_ = 0.197). Relatively low long‐distance dispersal capability and vitality of pollen contributed to high genetic differentiation among populations. Differences in the environmental conditions and long distance among populations further restricted gene flow. Structure clustering analysis uncovered intraspecific divergence between central and marginal populations. Migrate analysis found a high level of gene flow between these two intraspecific groups. Bayesian skyline plot detected population expansion of these two intraspecific groups. Network and phylogeny analysis of chloroplast haplotypes also found intraspecific divergence, and the divergence time was estimated to occur at about 55.86 Ma. Haplotype native to the Loess Plateau was more ancient, and multiple glacial refugia of sour jujube were found to locate at the Loess Plateau, areas adjacent to the Qinling Mountains and Tianmu Mountains. Species distribution model analysis found a typical contraction‐expansion model corresponding to the Quaternary climatic oscillations. In the future, the distribution of sour jujube may shift to high‐latitude areas. This study provides new insights for phylogeographic research of temperate plant species distributed in Central China and sets a solid foundation for the application of the scientific management strategy of *Z. jujuba* var. *spinosa*.

## Graphical abstract

Nucleotide variation and population differentiation of Ziziphus jujuba var. spinosa were relatively high and a central and marginal population structure was revealed. Multiple glacial refugia were found in central and east China. Population dynamic was found to conform to the contraction‐expansion model corresponding to the Quaternary climatic oscillations and may shift to high latitude areas under future climate conditions.

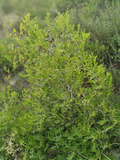

## 1 INTRODUCTION

The evolutionary history of plant species has emerged as a complex interaction of biogeography, climate change, and human forces (Feng et al., 2018; Paola et al., 2017). Phylogeography seeks to understand the comprehensive evolutionary history and distribution of organisms and has become a focus of evolutionary biology (Abbott & Comes, 2004; Riddle, 2010). Of particular importance to Chinese phylogeographic research, the Qinghai‐Tibetan Plateau (QTP; Gao et al., 2019; Khan et al., 2018; Qiu et al., 2011), Southwestern China (Du et al., 2017; Hou et al., 2018; Zheng et al., 2017), and Northern China (Bai et al., 2010; Zeng et al., 2016) have attracted more attention resulting from the substantial diversity of plant species, the topographically heterogeneous terrain and the complex climate conditions. These studies shed light on the interactions of geography, ecology, and climate in shaping the distribution and genetic pattern of plant species. However, phylogeographic research concerning Central China (such as the Loess Plateau and adjacent areas) has been rarely conducted (Wen et al., 2020). Some endemic species are narrowly and concentrated distributed in Central China, such as *Xanthoceras sorbifolia* Bunge (Zhu, 2016) and *Elaeagnus mollis* Diels (Du et al., 2020). Furthermore, the Quaternary glaciation cycles had little effect on this area, glaciers only formed in scattered areas of the Loess Plateau and lasted for a relatively short period (Zheng et al., 1998). Phylogeographic study of *E. mollis*, an endangered deciduous shrub species restricted distributed in the Loess Plateau, revealed multiple potential glacial refugia and allopatric divergence in Central China (Du et al., 2020).

*Ziziphus jujuba* var. *spinosa*, also known as sour jujube, is a deciduous shrub plant species belonging to *Ziziphus*, Rhamnaceae. *Z. jujuba* var. *spinosa* is widely distributed in the temperate region of China, especially in areas from the Loess Plateau to the Taihang Mountains, which corresponds to its climate and habitat optimum (Zhang et al., 2015; Zhao et al., 2021). It harbors significant ecological values and is typically used for soil and water conservation in Northern China as the high tolerance to drought and salt stress (Wang et al., 2018). The nutritional value of the fruit and the medicinal importance of the seed of sour jujube has kept its economic vitality in China for more than 2000 years (Qu, 1982). It is concluded that Chinese jujube (*Z. jujuba* Mill) is originated from sour jujube, and the evolutionary path may involve several different patterns (Liu, 1993; Peng, 1991). Genome‐resequencing SNP data have been utilized to investigate the genetic structure of sour jujube with Chinese jujube, and the clear separation of sour jujube and Chinese jujube, and the genome evolution and domestication history of sour jujube were highlighted (Guo et al., 2021; Huang et al., 2016; Shen et al., 2021). As the primitive ancestor of *Z. jujuba*, sour jujube is considered as a valuable gene pool for the genetic improvement of Chinese jujube (Zhang et al., 2015). Accompanied by the growth of the plantation of Chinese jujube and the overharvesting of the seeds, the distribution range of sour jujube is undergoing severe fragmentation and decrease (Gao et al., 2008). Previous research on sour jujube mainly focused on taxonomic classification, nutritional and medicinal ingredients, and responses to various abiotic stress (Kang et al., 2008; Li et al., 2017; Liu, 1993; Yan et al., 2017). The genetic diversity and genetic structure of sour jujube have not been well characterized, which hinders the comprehensive understanding and utilization of this important genetic resource. Zhang et al. (2015) investigated the genetic diversity and population structure of sour jujube using SSRs and found high level of genetic diversity (*H*_E_ = 0.659 and *H*_S_ = 0.674) and moderate differentiation among populations (*F*_st_ = 0.091, *R*_st_ = .068). However, the phylogeographic pattern and population demography of sour jujube corresponding to various climate conditions were still yet to be clarified. Phylogeography study of sour jujube may add acknowledgement of the species' evolutionary history in Central China and provide some clues for the potential migration between this area and other adjacent areas.

In the present study, single‐copy nuclear gene markers were utilized to investigate the genetic diversity and population structure of sour jujube distributed across China. Furthermore, chloroplast genome data was used to detect the phylogeographic history of sour jujube. The population dynamic of sour jujube responding to the Quaternary and future climatic changes was also documented. The aims of this study were as follows: (i) determine the genetic diversity and population structure of *Z. jujuba* var. *spinosa*, (ii) describe the phylogeographic history and the potential location of glacial refugia, and (iii) investigate the population dynamic of sour jujube in China. The present study will improve our knowledge of plant phylogeography in Central China and set a solid foundation for future management and utilization of this important germplasm resource.

## 2 MATERIALS AND METHODS

### 2.1 Population sampling and DNA extraction

**FIGURE 1:**
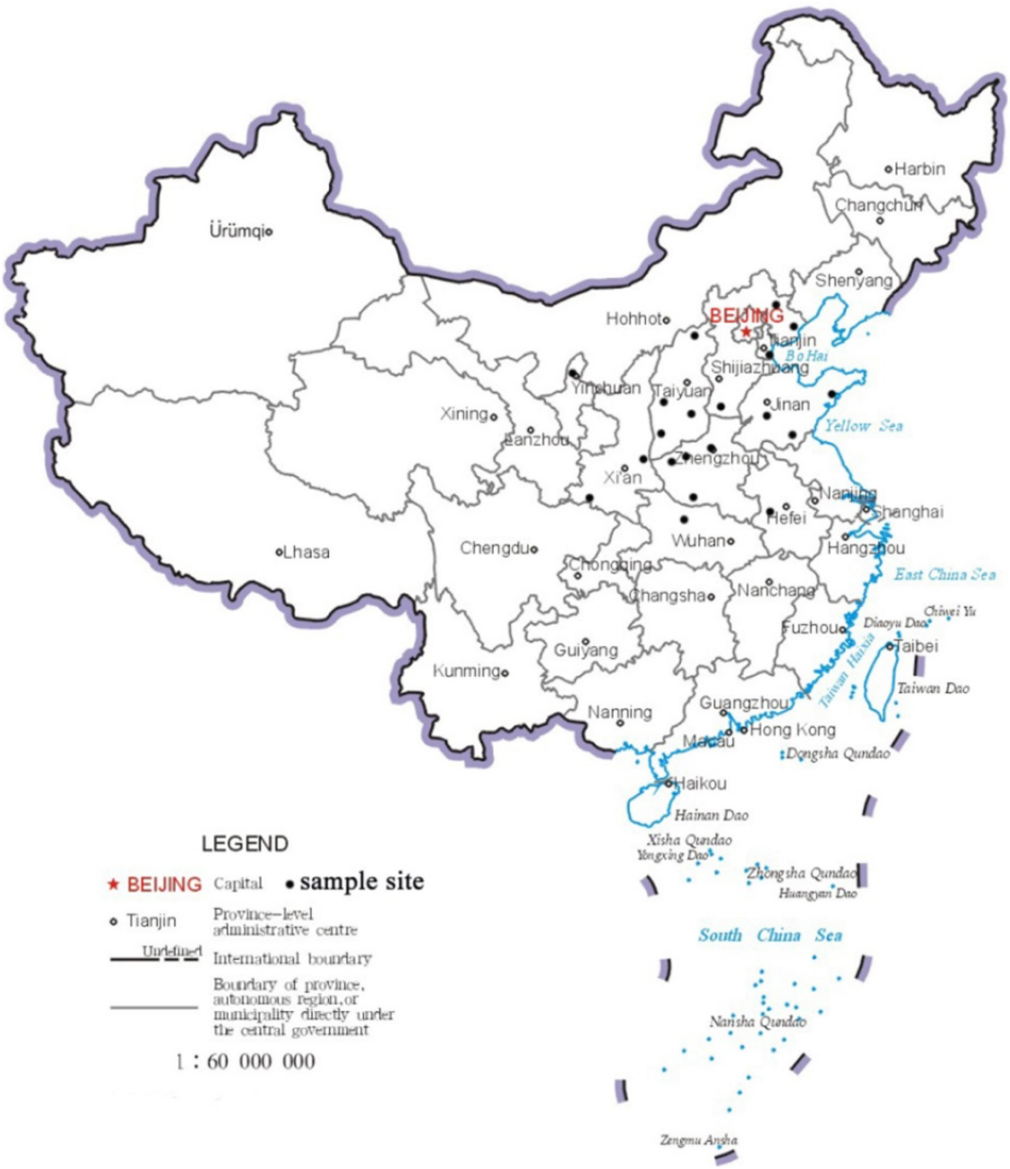
Sampled populations of sour jujube in this study

In this study, 328 individuals were sampled from 21 sour jujube populations distributed in China. The geographic information of the populations sampled in this study was illustrated in Figure 1 and Table S1. We also sampled one Chinese jujube (*Z. jujuba* “Hupingzao”) and one *Z. mauritiana* individual as outgroup in the following analysis. Individuals sampled in the same population were at least 100 m apart. Total DNA was extracted for all sampled individuals from silica gel‐dried leaves using the modified cetyltrimethyl ammonium bromide (CTAB) method.

### 2.2 Locus selection, amplification and sequencing

Single‐copy nuclear gene markers have been widely used in plant population genetic studies, resulting from their cross‐utility, high variable, and easily processed features (Du et al., 2015; Hou et al., 2018; Liu et al., 2016; Wang et al., 2019). In this study, we selected five single‐copy nuclear gene markers developed in our previous research for amplification and sequencing of all the samples (Hu, Du, Wang, & Han, 2021). Detailed information about the locus was listed in Table 1. Polymerase chain reaction (PCR) amplification followed the protocols established in Hu, Du, and Han (2021). Direct sequencing was performed on an ABI 3730XL DNA analyzer (Applied Biosystems) using the same primers that were used for amplification.

**TABLE 1:** Primers for the single‐copy nuclear gene markers used in this study

Locus	Primer sequences (5′–3′)	Gene annotation	Ta (°C)
SZ2	F:TGGTACAGGATCTACAATTC R:CCTGACTTTCTAATTGCTTC	myb‐like protein X	52
SZ11	F:ATGGCTTTTGCTTGCCTCTC R:GTGCATTCGGGTCATCAATG	UDP‐N‐acetylmuramoyl‐L‐alanyl‐D‐glutamate‐‐2,6‐diaminopimelate ligase MurE homolog	53
SZX2	F:GCTACTCGCTCTGGTTTCCAT R:GAAGAATCCTTGCCGGTTCAG	Trichohyalin	54
SZX12	F:GCCCTTTCGCAAAGCTTTCTT R:CCCAACACTGAGATTACTGGAG	Transcription termination factor MTEF18	54
SZX13	F:AAATGGAAGCGGCCTAGTGA R:TCCAGGAGTTTCCTCAGAGTC	DNA repair endonuclease UVH1	54

### 2.3 Genetic diversity and the neutral test

The assembled contigs of each individual were aligned using Clustal X (Thompson, 1997) and refined manually in Bioedit (Hall, 1999). The number of segregating sites (S), nucleotide diversity parameters, *π* (Nei, 1987) and Watterson's *θ*_w_ (Watterson, 1975), and the minimum number of recombination events (*R*_m_) were analyzed for the five loci using Dnasp 5.10.0 (Librado & Rozas, 2009). We also calculated Tajima's *D* (Tajima, 1989), Fu and Li's D* and F* (Li & Fu, 1993) for each locus using Dnasp 5.10.0 to test how well the data conformed to the neutral model of evolution. Furthermore, we performed a standard multilocus Hudson‐Kreitman‐Aquadé (MLHKA) test (Hudson et al., 1987) to test for selection. The sequences of *Z. mauritiana* were used as an outgroup for the MLHKA test.

### 2.4 Genetic differentiation

The distribution of nucleotide variation among populations at each locus was assessed using AMOVA (analysis of molecular variation) implemented in Arlequin 3.5 (Excoffier et al., 2005) with significance testing evaluated using 1000 permutations of the original data. Genetic variation was hierarchically partitioned into among populations within species and within populations. Pairwise Wright's fixation index, *F*_st_ (Wright & Maxson, 1968), was used to measure population differentiation Furthermore, to assign the sampled individuals to different genetic clusters (*K*), genetic structure analysis was performed in Structure 2.3.4 (Falush et al., 2003) with these five loci. Ten independent runs for each possible value of *K* from 1 to 10 were performed with a burn‐in of 500,000 following 1000,000 MCMC iterations with an admixture model and correlated allele frequencies. The most likely value of *K* based on the negative natural log‐likelihood of the data (Ln*P*(*K*)) and Δ*K* was calculated using Structure Harvester (Campana et al., 2011; Evanno et al., 2005). Distruct 1.1 (Rosenberg, 2004) was used to create and visualize the population bar plots.

### 2.5 Chloroplast genome analysis

To understand the phylogeography history of sour jujube in China, we sequenced and assembled the complete chloroplast genome of 21 individuals (randomly chose one individual from each sampled population). The chloroplast haplotypes were assigned using Dnasp 5.0, and the median‐joining (MJ) haplotype network was generated in Popart 1.7 (Leigh & Bryant, 2015) with the chloroplast genome sequence of *Z. mauritiana* (NC_037151) as outgroup. The divergence time of the chloroplast haplotypes was detected with BEAST 1.10 (Suchard et al., 2018). Complete chloroplast genome sequences of *Z. jujuba* (NC_030299), *Z. mauritiana*, *Z. spina‐christi* (NC_037152), and *Morus indica* (NC_008359) were utilized as outgroups. A likelihood ratio test (LRT; Felsenstein, 1988) of the data implemented in MEGA6 (Tamura et al., 2013) rejected the molecular clock hypothesis with *p* < .01. A GTR nucleotide substitution model determined by jModeltest 3.7 (Posada, 2008) and a Yule speciation process were used. Using complete genome sequences, Liu et al. (2014) revealed that *Z. jujuba* diverged from other Rosales species at about 79.9 Ma (million years ago, 95% HPD: 55.7–99.4 Ma) and this time with a standard deviation of 5 Ma was used to constrain the maximum root age of the phylogeny. Four independent MCMC replications were performed, and each replication was run for 100,000,000 steps and sampled every 1000 steps. The first 25% of the sampled trees were discarded as burn‐in. The program Tracer 1.7.1 (http://tree.bio.ed.ac.uk/software/tracer) was used to confirm that the runs had reached a stationary distribution with sufficient mixing. Figtree 1.3 (http://tree.bio.ed.ac.uk/software/figtree/) was used to visualize the phylogenetic tree.

### 2.6 Coalescent‐based population demographic history analysis

To reconstruct the demographic history of sour jujube and the two intraspecific groups revealed in Structure analysis (see below) over time, the historical demography was inferred from Bayesian skyline plot analyses (BSP) implemented in BEAST 1.10 (Suchard et al., 2018). This coalescence‐based approach utilized a standard MCMC sampling procedure to evaluate the posterior probability distribution of effective population size backward until the time to the most recent common ancestor of the sampled sequences was reached. All the five loci were used for this analysis. The best‐fit substitution model of the concatenated sequence was determined by jModeltest 2 (Posada, 2008). Independent MCMC analysis was run for 1 × 10^8^ steps, sampling every 100 steps and discarding 25% of sampled trees as burn‐in. For each group, multiple independent analyses were performed with different random seeds to test for convergence, and results of replicate runs were pooled using LogCombiner1.10.4, and skyline plots were visualized with Tracer 1.7.1 (http://tree.bio.ed.ac.uk/software/tracer).

### 2.7 Gene flow analysis

An MCMC maximum likelihood method was utilized to estimate gene flow between two intraspecific groups using Migrate 4.4.3 (Beerli, 2001). Two important population genetic parameters, *θ* (four times effective population size multiplied by mutation rate per site per generation) and *M* (immigration rate divided by the mutation rate) were calculated with the implemented *F*_st_ estimations. Five independent runs by 10 short chains of 5000 steps and 3 long chains of 50,000 steps were run. Genealogies with a sampling increment of 100 and 10,000 burn‐in were recorded.

### 2.8 Landscape genetics

The biogeographic boundaries were calculated by Monmonier's maximum difference algorithm in Barrier 2.2 (Manni et al., 2004) based on the geographic and genetic distance matrix. Permutation and bootstrap tests were conducted with 1000 replicates. Furthermore, to evaluate the effect of geography on genetic differentiation, the correlation between geographic distance (GeoD) and genetic distance (GenD) among populations was calculated by the Mantel test (Mantel, 1967) using Vegan package implemented in R (Simpson et al., 2010).

### 2.9 Impact of environmental factors on genetic structure (isolation by environment)

In order to evaluate the effect of present environmental conditions on the observed pattern of the genetic structure of sour jujube, the correlation between GenD and environmental distance (EnvD) among these sampled populations was calculated by Mantel test using Vegan package implemented in R. Twenty‐three environmental variables of the 21 sampled populations were determined for the current climate layers as used in species distribution modeling analysis (see below). EnvD matrix was computed based on the population score on each bioclimatic variable.

### 2.10 Species distribution modeling

Twenty‐three environmental variables with 30 s spatial resolution were used in species distribution modeling (19 bioclimatic variables and elevation data were directly downloaded from WorldClim dataset [www.worldclim.org], soil variable was downloaded from the National Geomatics Center of China, slope and aspect were generated from elevation variable through surface analysis in Arcgis 10.5 [ESRI, Environmental Systems Research Institute]). These environmental variables were held in a GIS as ESRI grids using China Map Grid coordinates. The multi‐collinearity test was conducted by using the Pearson correlation coefficient (*r*) to examine the cross‐correlation among the variables and variables with cross‐correlation coefficient value |*r*| ≥ .8 were randomly discarded one and kept the other to minimize the negative influence on modeling. Finally, 10 environmental variables including Bio3 (iso‐thermality [Bio2/Bio7] × 100), Bio7 (temperature annual range[Bio5‐Bio6]), Bio11 (mean temperature of the coldest quarter), Bio15 (SD of humidity seasonality), Bio18 (precipitation of the warmest quarter), Bio19 (precipitation of the coldest quarter), elevation, slope, aspect, and soil were kept and utilized in species distribution modeling (Table S2).

Species distribution modeling was performed with Maxent 3.4.4 (Phillips et al., 2006; Phillips & Dudík, 2008), which used the maximum entropy method to model species' distribution. Past (including the Last Interglacial, the Last Glacial Maximum, and middle Holocene), present day, and future (including 2050s and 2070s under RCP2.6 [Representative Concentration Pathways], RCP4.5, and RCP8.5) niche models for sour jujube were generated. Distribution data of sour jujube was collected based on the present study and retrieved in National Plant Specimen Research Center (http://www.cvh.ac.cn/) and distribution information listed in Zhang et al. (2015) and Zhao et al. (2021). A total of 519 distribution data were collected, and R package “spThin” was utilized to remove the samples that clustered within 10 km to reduce the sampling deviation impact (Aiello‐Lammens et al., 2015), and delete redundant data. To reduce sampling deviation, only one distribution point was kept for each grid (10 × 10 km), and finally 253 effective distribution records were used in the following analysis (Table S3). In Maxent analysis, 80% of the distribution localities were used to train the model and 20% were randomly selected to test the model. 10 replicate runs were performed, and replicated run type was set to cross‐validate. The random seed was used to ensure consistency in the statistical output between runs. Model performance was evaluated using the area under the receiver operating characteristic curve (AUC). The value of AUC varies between 0.5 and 1, and a higher value means higher model accuracy, although in practice the maximum possible value of AUC is often <1 (Phillips et al., 2006). The Jackknife procedure was used to assess the importance of the variables contributing to the model. Model predictions were visualized in Arcgis 10.5 (ESRI). Based on the definition of species' suitable distribution area in WCRPCMIP6 (World Climate Research Program Coupled Model Intercomparison Project Phase 6, https://esgf‐node.llnl.gov/projects/cmip6/), the value of distribution possibility was reclassified into three hierarchy in Arcgis 10.5: 0–0.33 means unsuitable distribution area; 0.33–0.66 means suitable area and 0.66–1.0 means higher suitable area.

## 3 RESULTS

### 3.1 Nucleotide variation and neutrality test

We successfully sequenced 5 single‐copy nuclear gene markers from all the individuals sampled in this study. As shown in Table 2, the length of the single‐copy nuclear gene markers ranged between 190 and 846 bp. The average number of segregating sites and haplotype diversity were 37 and 0.857, respectively. A total number of 237 SNPs (single nucleotide polymorphism) was detected through the five single‐copy nuclear gene markers. Two important nucleotide variation parameters, *π* and *θ*_w_, reached 0.000720 and 0.00925, indicating the high level of nucleotide variation of sour jujube. The average number of recombination events throughout the single‐copy nuclear gene markers was 8, which indicated that the sour jujube genome underwent frequent inter‐chromosome fusions and segmental duplication events (Liu et al., 2014). Results of Tajima's *D*, Fu, and Li's D* and F* showed significant departure from neutrality at some loci. The result of the MLHKA test showed no significant departure from neutral expectation (*χ* ^2^ = .010, *p* = .9200), indicating that the significant results of Tajima's *D*, Fu, and Li's D* and F* detected in these loci may result from the presence of recombination within loci or the population structure (Ramos‐Onsins & Rozas, 2002; Zheng & Ge, 2010).

**TABLE 2:** Result of nucleotide variation of five single‐copy nuclear gene markers and neutrality tests

Locus	*L*	*S*	*H*_d_	*π*	*θ*_w_	*D*	D*	F*	*R*_m_	*N*_m_
SZ2	309	24	0.611	0.00619	0.01164	−1.16927	−4.95722**	−4.07025**	3	1.16
SZ11	846	82	0.940	0.00750	d0.01694	−1.59189	2.18531**	0.28719	17	0.67
SZX2	190	28	0.704	0.00746	0.02347	−1.75806*	−0.27874	−1.13229	3	1.93d
SZX12	648	43	0.923	0.00503	0.01124	−1.50814	−4.08123**	−3.46958**	9	1.09
SZX13	730	60	0.929	0.00765	0.01321	−1.17321	−3.45451**	−2.81122*	8	0.84
Mean	—	37	0.857	0.00720	0.00925	—			8	1.33

* *p* < .05.

** *p* < .01.

### 3.2 Population structure and genetic differentiation

AMOVA analysis was used to investigate the overall distribution of genetic variation (Table 3). Variation among populations ranged from 9.06% at locus SZX2 to 27.96% at locus SZ11 and was significant (*p* < .001). Most of the total variation was derived from variation within populations (72.04%–90.94%). The average *F*_st_ was 0.197, indicating the relatively high level of genetic differentiation among populations.

**TABLE 3:** AMOVA and *F*_st_ analysis of *Ziziphus jujuba* var. *spinosa* populations in China

Locus	SZ2	SZ11	SZX2	SZX12	SZX13	Mean
Among populations (%)	24.37	27.96	9.06	15.84	21.28	19.70
Within populations (%)	75.63	72.04	90.94	84.16	78.72	80.30
*F*_st_	0.243	0.279	0.090	0.158	0.212	0.197

**FIGURE 2:**
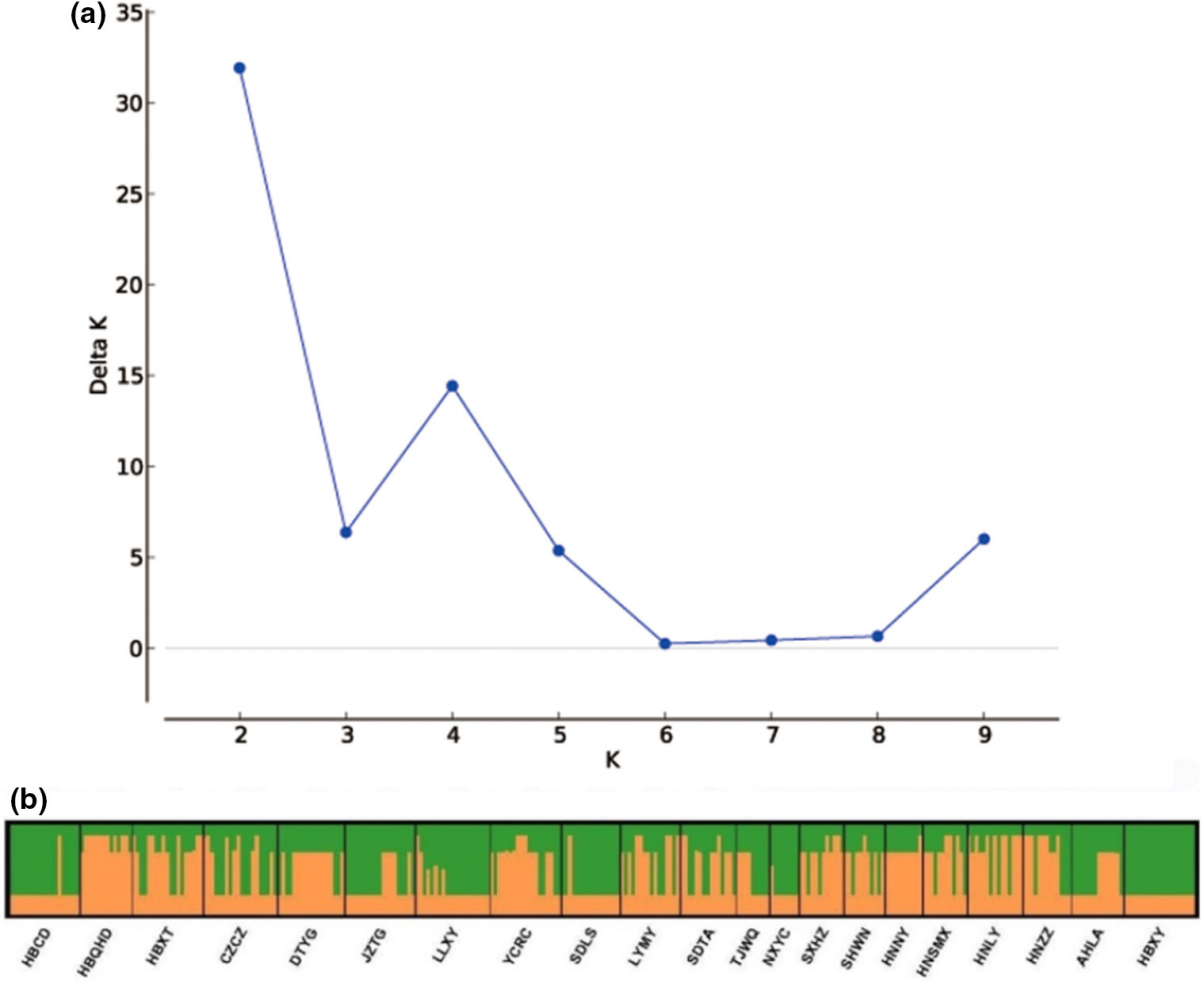
Result of structure analysis (a: result of Δ*K*, b: genetic composition of *Ziziphus jujuba* var. *spinosa* individuals)

**FIGURE 3:**
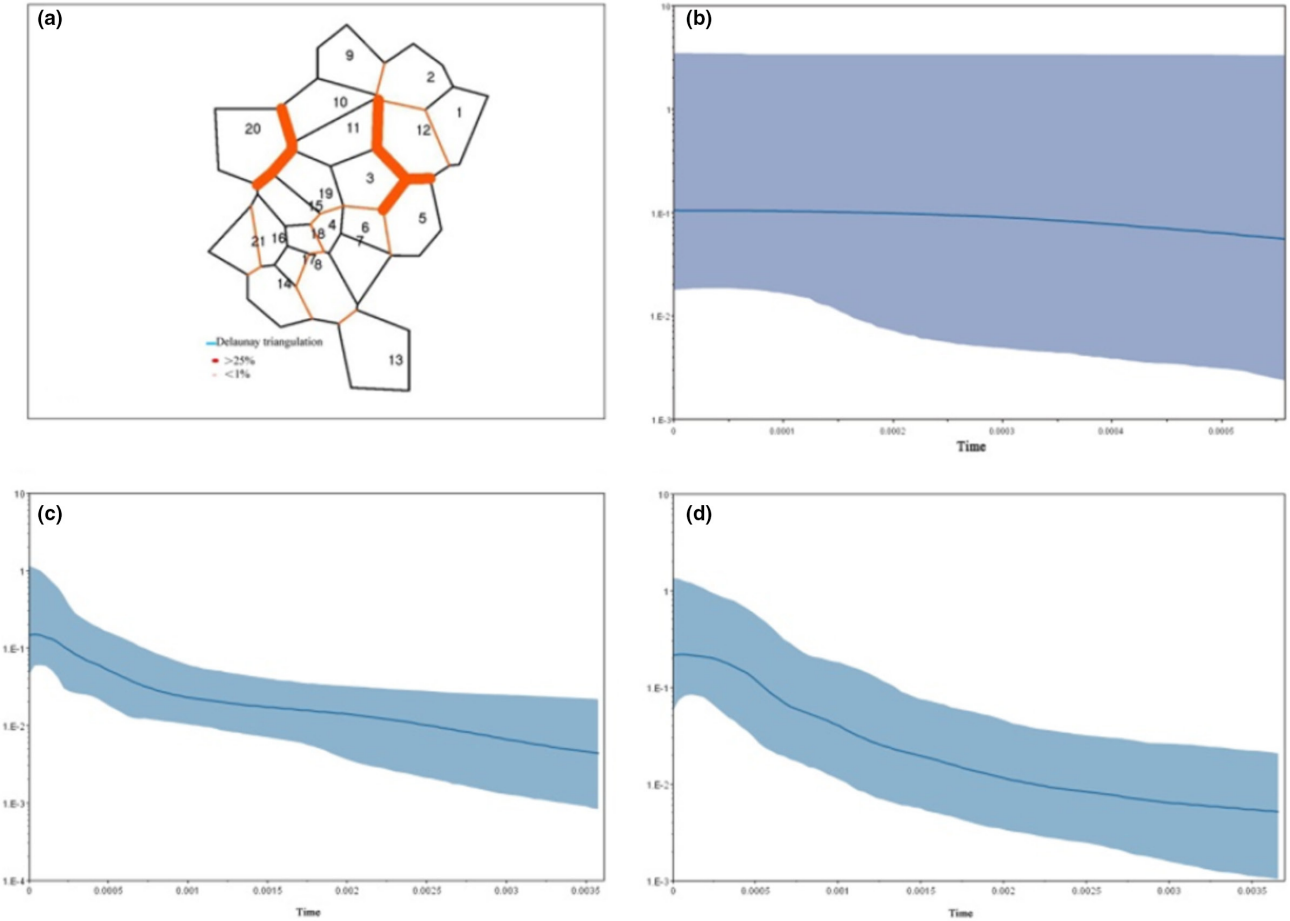
Result of barrier analysis and geographic location of the genetic barrier was indicated by thick red lines (a) Bayesian skyline plots for the three groups, showing effective population size as a function of time. The upper and lower limits of light blue trend represented the 95% confidence intervals of HPD analysis. (b) Total sour jujube populations; (c) central populations; (d) marginal populations

Following the method of Evanno et al. (2005), two significant genetic clusters were detected in Structure analysis (Figure 2). According to the estimated ancestry, most sampled individuals showed an average inferred major membership proportion higher than 0.60, which indicated that they can be classified as belonging to one of the two distinct genetic clusters. Among the populations in genetic cluster 1, most were distributed in the area from the Lose Plateau to the Taihang Mountains (83.33%), which was the distribution center of sour jujube in China. Therefore, these populations in cluster 1 were referred to as central populations in the following analysis. Populations in genetic cluster 2 mainly consisted of populations that were distributed far away from the central area (55.56%), and they were referred to as marginal populations. Furthermore, some populations may be classified as hybrid populations of the two genetic clusters, with the proportion of membership lower than 0.6 in both clusters (Zhang et al., 2015). These populations were mainly distributed in the adjacent area of central and marginal populations (Table S4). Migrate 4.4.3 was employed to estimate the historical gene flow between the central and marginal populations. Results showed that the effective population size of the central population was about 1.5 times that of the marginal population. Gene flow between these two groups was asymmetric (Table 4). Mantel test of relationship between GeoD, EnvD and GenD showed significance correlation (GeoD/GenD, *r* = .2619, *p* = .02; EnvD/GenD, *r* = .3873, *p* = .001). Analyses of landscape genetics using Barrier 2.2 identified four significant barriers among sampled populations (.25 < *p* < .41; Figure 3a). Most of the barriers appeared between the central and marginal populations, which further proved the intraspecific divergence within sour jujube. BSP analysis revealed that the effective population size of sour jujube and the two intraspecific groups underwent substantial expansion over time (Figure 3b–d).

**TABLE 4:** Result of historical gene flow between population clusters

	*θ*	*M*	
		CP → MP	MP → CP
Central population (CP)	0.01497 (0.00940–0.02160)	1696.7 (1432–1978.7)	
Marginal population (MP)	0.00943 (0.00113–0.02)		499.3 (64–301.3)

### 3.3 Chloroplast genome analysis

**FIGURE 4:**
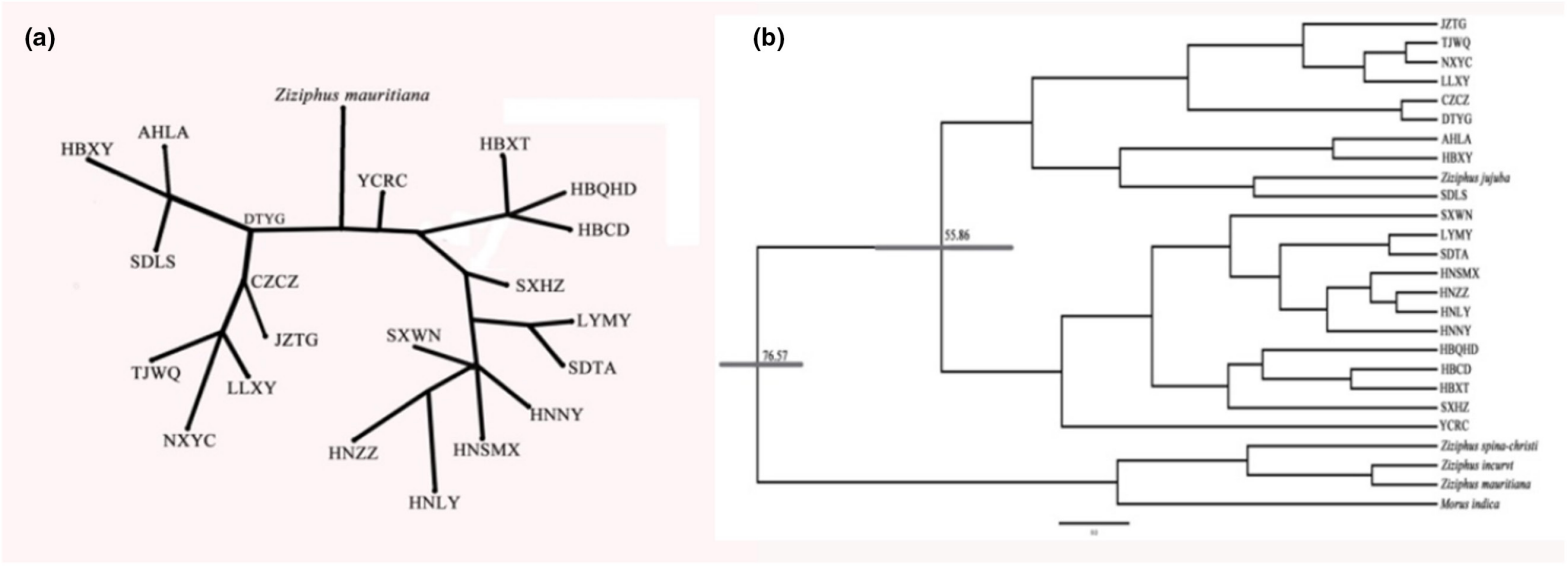
Network and BEAST analysis of sour jujube chloroplast genome sequences (a: network analysis; b: BEAST result. Number next to nodes indicated divergence time, and dark bar indicated 95% HPD)

We successfully sequenced and assembled the complete chloroplast genome of 21 sour jujube individuals randomly selected from each sampled population. The length of the complete chloroplast genome varied between 159,399 and 161,279 bp, and GC content ranged from 36.51% to 37.30%. Detailed information about the sour jujube chloroplast genome was listed in Table 5. A total of 21 chloroplast haplotypes (H1–H21) were designated and the outgroup *Z. mauritiana* contained a private haplotype (H22). The MJ network of the chloroplast haplotypes showed that the chloroplast haplotype native to population YCRC was ancestral, which was also supported by BEAST analysis (Figure 4). Both network and BEAST analysis revealed two intraspecific lineages within sour jujube, which was consistent with the result of single‐copy nuclear gene marker Structure analysis. The divergence time of these two intraspecific lineages was dated back to about 55.86 Ma (95%HPD: 31.82–79.37 Ma). Furthermore, in BEAST analysis, chloroplast genome of Chinese jujube occupied a terminal position in the phylogenetic tree, which indicated that Chinese jujube may originate or artificially domesticate from sour jujube. The exact domestication process may need further research with more data.

**TABLE 5:** Detailed information about the chloroplast genome of 21 sour jujube individuals

Individual	Length (bp)	GC content (%)	LSC (bp)/GC(%)	SSC (bp)/GC(%)	IR (bp)/GC(%)
HBCD19	160,876	36.92	88,526/34.7	19,392/30.9	26,479/42.6
HBQHD5	160,730	36.69	88,690/34.5	19,364/30.8	26,338/42.7
HBXT5	160,691	36.86	88,411/34.7	19,358/30.9	26,461/42.7
CZCZ1	160,750	36.86	88,378/34.7	19,378/30.9	26,497/42.6
DTYG9	160,795	36.84	88,481/34.7	19,356/30.8	26,479/42.6
JZTG11	160,764	36.69	88,737/34.5	19,353/30.8	26,337/42.7
LLXY7	159,754	36.89	87,701/34.8	19,107/30.8	26,470/42.9
YCRC14	161,259	36.74	88,945/34.5	19,356/30.9	26,479/42.6
SDLS10	161,211	36.75	88,897/34.6	19,356/30.9	26,478/42.6
LYMY19	160,705	36.70	88,674/34.5	19,359/32.4	26,336/43.0
SDTA10	161,278	36.77	88,964/34.6	19,356/30.9	26,479/42.6
TJWQ2	161,100	36.98	88,862/34.6	19,366/30.9	26,436/42.6
NXYC4	161,062	36.80	88,746/34.6	19,358/30.9	26,479/42.6
SXHZ9	161,279	36.76	88,888/34.6	19,361/30.9	26,515/42.6
SXWN9	160,918	36.79	88,640/34.6	19,356/30.9	26,461/42.7
HNNY2	159,994	36.99	87,751/34.9	19,259/31.0	26,492/42.6
HNSMX5	160,347	36.93	87,995/34.8	19,368/30.9	26,492/42.6
HNLY9	159,399	37.30	87,244/35.5	19,127/31.1	26,514/42.6
HNZZ14	161,100	37.01	88,862/34.6	19,361/30.9	26,436/42.6
AHLA1	160,668	36.51	88,573/34.2	19,339/30.5	26,378/42.7
HBXY14	161,080	36.85	88,751/34.6	19,361/30.9	26,484/42.7

Abbreviations: LSC, long single‐copy region; SSC, short single‐copy region; IR, inverted repeat sequence; LGM, Last glaciation maximum.

### 3.4 Distribution model

**FIGURE 5:**
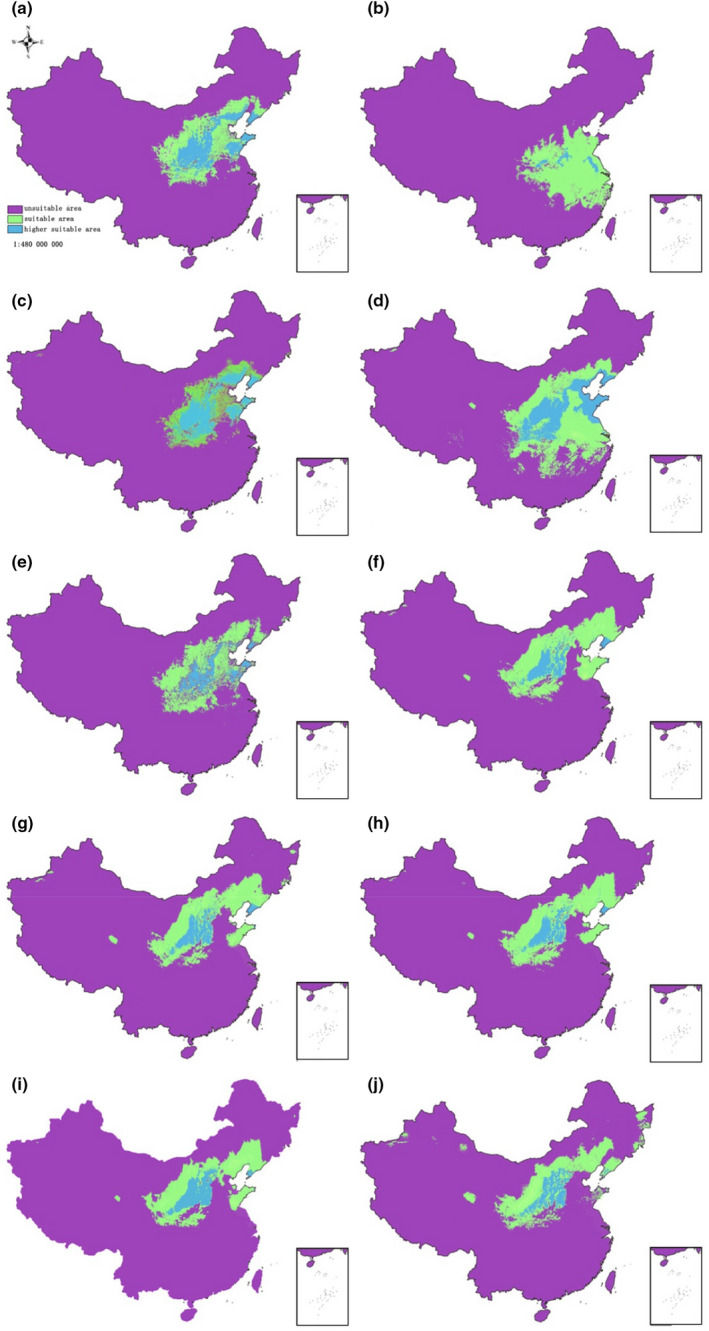
Distribution dynamic of *Ziziphus jujuba* var. *spinosa* in different periods (a: present; b: last glacial maximum; c: middle holocene; d: last interglacial; e: 2050s, RCP2.6; f: 2050s, RCP4.5; g: 2050s, RCP8.5; h: 2070s, RCP2.6; i: 2070s, RCP4.5; j: 2070s, RCP8.5)

Using 10 environmental variables, the population distribution dynamic of sour jujube in different periods in China was modeled with Maxent (Figure 5). The result showed that the average value of the AUC of training data in different periods was 0.943 while that of the test data were 0.932, meaning higher accuracy of the distribution model (Table S5). The contribution rate of Bio11 was the highest (35.4%), and soil was the least (0.1%) to the model in the Jackknife procedure (Table S6). Because the average contribution rate of Bio11, Bio18, Bio7, and elevation to the Maxent model was higher than 10% and the accumulated contribution rate of these four variables was >80%, these four variables were classified as dominant variables affecting the distribution of sour jujube in different periods.

The present potential distribution of sour jujube predicted in Maxent was highly consistent with the actual species' distribution (Wu, 1982). During the LGM, the range of sour jujube contracted considerably and showed a southward range shift. The proportion of the higher suitable area decreased from 2.08% at present to 0.80%, and the species was probably restricted to areas between the south of the Loess Plateau and the north of the Qinling Mountains, and areas adjacent to the Tianmu Mountains in East China. During the MH, the total suitable area of sour jujube increased from 9.79% in LGM to 11.45% and the higher suitable area mainly located in Shandong, Hebei, Shanxi, and Shaanxi provinces. In LIG, with the improvement of the climate, the area of total suitable area of sour jujube increased to 17.84% and the higher suitable area mainly located in Central China (from the Loess Plateau to Shandong Peninsula) and around Bohai gulf. In the future (2050s and 2070s), the area of the total suitable area of sour jujube decreased but that of the higher suitable area increased. The distribution range of sour jujube clearly showed a northward shift to high‐latitude areas in future environmental conditions (Figure 5 and Table S6).

## 4 DISCUSSIONS

### 4.1 Nucleotide variation of sour jujube in China

With the development of sequencing and analytical technology, genome data, such as genome‐resequencing and RAD‐seq (Restriction site‐associated DNA sequencing) data containing tens of thousands of SNPs have been widely applied in population genetic research (Lange et al., 2021; Mu et al., 2020). Using genome‐resequencing SNP data, the nucleotide variation of sour jujube was found to be higher than that of Chinese jujube (Guo et al., 2021; Huang et al., 2016). Though the number of SNPs in the present study (237 SNPs in five single‐copy nuclear gene markers) was small, but the basic population genetic information of sour jujube was revealed to some extent and added to our acknowledgement of this important plant species. Future genome data may apply in the genetic‐related study of sour jujube, such as the domestication history of sour jujube and the underlying mechanism for metabolite differences between sour jujube and Chinese jujube.

Although nucleotide variation of sour jujube at the locus level varied substantially, sour jujube distributed in China showed a relatively high level of nucleotide variation (*π* = 0.00720, *θ*_w_ = 0.00925). Nucleotide variation of sour jujube was higher than that of other species distributed in China, such as *Populus davidiana* (*π* = 0.00440, *θ*_*w*_ = 0.00750; Du et al., 2015), *Tamarix austromongolica* (*π* = 0.002 59; Wen et al., 2020) and *Pugionium cornutum* (*π* = 0.00532; Wang et al., 2013), and even comparable to some annual plant species such as *Hordeum vulgare* ssp. *spontaneum* (*θ*_w_ = 0.0109), *Zea mays* (*π* = 0.0086–0.0133, *θ*_w_ = 0.0094–0.015; Wright & Gaut, 2005), *Arabidopsis thaliana* (*π* = 0.0080–0.0165, *θ*_w_ = 0.0049; Ramos‐Onsins et al., 2010). This was consistent with research on the genetic diversity of sour jujube using other genetic markers (i.e., SSR and RAMP; Zhang et al., 2015; Zhang et al., 2014). Furthermore, genome‐resequencing data also showed that the nucleotide diversity of sour jujube was higher than that of some perennial crops, such as peach (Li et al., 2019), but lower than that of date palm (Hazzouri et al., 2015), apple (Duan et al., 2017), and pear (Wu et al., 2018). The variation between this study and other research was partly attributed to the different parts of the genome analyzed.

A series of factors may contribute to the nucleotide variation of plant species, including representative sampling, natural selection, mating system, and demographic history (Wright & Gaut, 2005). Representative sampling can be ruled out to a great extent because the sampling area of this study covered the main distribution range of sour jujube in China, especially the areas from the Loess Plateau to the Taihang Mountains. Natural selection can also be excluded because the test statistics indicated no significant deviation from neutrality. Sour jujube was obligated to outcross and inbreeding seldom occurred in natural populations (Zhang, 2013), which contributed to a relatively high recombination rate and effective population size, and ultimately to a high level of nucleotide variation (Charlesworth, 2003). Considering how living organisms responded to global climate fluctuations, specially the glacial–interglacial cycles in the Quaternary (Qiu et al., 2011), we primarily speculated that sour jujube may undergo a process of population contraction and expansion during the evolutionary history. But the BSP analysis revealed that the effective population size of sour jujube underwent a continuous increase. This paradox can be clarified that the Quaternary glaciation had little effect on areas in Central China, especially the Lose Plateau (Zheng et al., 1998). In other central distribution areas of sour jujube, such as the Taihang Mountains, the population of plant species often migrated to high‐altitude areas to prevent severe bottlenecks from occurring, and maintained a higher effective population size (Hewitt, 2000). Consequently, the mating system and complex population dynamics have acted to retain a high level of genetic diversity in sour jujube distributed in China.

### 4.2 Genetic structure of sour jujube in China

In the present study, AMOVA analysis revealed that most of the total variation was attributed to variation within populations and a high level of genetic differentiation was detected (*F*_st_ = 0.197, Table 3). This indicated that gene flow among populations was restricted to a relatively low level. Zhang et al. (2013) investigated the genetic structure of 3 sour jujube populations along the Yellow River and found a high level of gene flow and a low level of genetic differentiation among these populations. They speculated that seed dispersal depending on the water flow of the Yellow River may accelerate gene flow among these populations. They further investigated the genetic structure of 34 sour jujube populations and also found moderate differentiation (*F*_st_ = 0.091; Zhang et al., 2015). The populations sampled in Zhang et al. (2015) were mainly distributed along the reach of some rivers, such as the Yellow River, the Jing River and the Luo River, seed dispersal by the river over long distance was the most probable explanation for gene exchange. However, there was no obvious connection among these populations sampled in the present study. Furthermore, the Mantel test revealed a significant correlation between geographic, environmental distance, and genetic distance. Differences in the environmental conditions and long distance resulted in a relatively high level of genetic differentiation among the sampled populations in this study. The long‐distance dispersal capability and the vitality of sour jujube pollen were relatively low (Shao et al., 2020). In general, it appears that forces such as isolation and adaptation that tend to increase genetic differentiation have been much stronger than homogenizing forces such as gene flow (Savolainen et al., 2007). Our results revealed a central, namely the Loess Plateau‐Taihang Mountains populations, genetic group surrounded by marginal populations that exhibited deep phylogeographic divergence as revealed in Network and phylogenetic analysis. The Migrate analysis on group‐level revealed a relatively high level of gene flow between these two intraspecific groups (Table 4). The existence of putatively hybrid populations may increase the estimation of gene flow. Furthermore, megafauna capable of long‐distance dispersal of seed cannot be completely ruled out, which played a certain role in shaping the genetic structure of some plant species (Feng et al., 2018). It is not yet possible to untangle natural gene flow over evolutionary time and recent human‐mediated gene flow to determine what traits characterized sour jujube population structure. Our analysis increased knowledge of the genetic diversity and population structure of sour jujube in China, revealed the potential geographic location of different gene pools, and could help to rationalize and prioritize reservoirs of genetic diversity.

### 4.3 Population demography of sour jujube in China

Zhao et al. (2021) compared 48 parameter combinations in R package ENMeval and Biomod2 to predict the distribution demography of sour jujube and found the Maxent model was optimal. In the present study, Maxent analysis yielded relatively accurate results that could be utilized to interpret the population demography of sour jujube corresponding to various environmental conditions in different periods. It was found that Bio11 contributed the most to the Maxent modeling, which reflected that low temperature was the main factor limiting the distribution of sour jujube. Sour jujube was not sensitive to soil conditions and its tolerance to barren soil was high (Wang et al., 2018; Zhao, 2016), which resulted in the lowest contribution of soil to Maxent modeling. The effects of Quaternary glaciation‐interglaciation cycles on the distribution pattern of plant organisms in the Northern Hemisphere have been substantially documented (Hewitt, 2004; Qiu et al., 2011). It has been proposed that temperate forests retreated southward to approximate 30°N during the Quaternary glacial periods based on paleovegetation data from East Asia and the populations located in northern areas must have recolonized from southern glacial refugia (Qian & Ricklefs, 2001). The paradigmatic contraction–expansion model of the latitudinal shift of temperate plant species responding to Quaternary climate oscillations has been drawn based on numerous phylogeographic surveys (Gonzales et al., 2008; Lafontaine et al., 2010). During the LGM, it was found that the distribution of sour jujube in China illustrated a typically southward retreat to about 30°N, and the higher suitable area mainly located in areas between the southward of the Loess Plateau and the northward of the Qinling Mountains, and areas adjacent to the Tianmu Mountains in East China. As mentioned above, glaciers only developed in southern scattered areas of the Loess Plateau and lasted for a relatively short time (Zheng et al., 1998). The mountainous areas were affected little by the Quaternary glacier and the climatic condition was relatively stable in the high altitudinal area. Therefore, appropriate climate conditions in these regions can serve as the highly suitable areas for sour jujube to survive the glaciation. During MH and LIG, the distribution of sour jujube showed a northward recolonization and expansion resulting from the improvement of climatic conditions (Yu et al., 2000; Zhao & Piperno, 2000). In the future (2050s and 2070s under 3 RCP conditions), the distribution of sour jujube in China showed northward migration to high latitudinal areas. Compared with the present distribution, the area of the higher suitable area increased but that of the total suitable area decreased. This was consistent with the research that under warming climate conditions, the distribution area of plant species could reduce and shift to high latitudinal areas (Ji et al., 2020; Walther et al., 2005). By simulating and predicting the distribution range of sour jujube in different periods, the historical causes of its formation and its future population dynamic responding to climate changes were deduced. Awareness of the possible effect of past climatic changes on the current population may provide insight into this species' future range dynamics in the light of climatic changes and be useful for germplasm management strategies (Paola et al., 2017; Ren et al., 2017).

### 4.4 Phylogeographic history of sour jujube in China

The effects of Quaternary climatic oscillations on the phylogeographic structure of species in the mid‐ to high‐latitude regions of Europe and North America (Emerson et al., 2010; Ren et al., 2017), high‐altitude areas (French et al., 2019; Gao et al., 2019; Khan et al., 2018), and Northern China (Hou et al., 2018; Zeng et al., 2016) have already been substantially documented. However, few studies have focused on the phylogeographic history of species native to temperate China. Therefore, the study presented here provided an opportunity to uncover the detailed population demographic history of a widely distributed plant species in temperate China and to better understand the processes playing roles in shaping the distribution pattern.

The fossil record was the primary clue to detect the distribution history of plant species. However, the fossil record for *Ziziphus* was not as complete or reliable as that of other species in Rhamnaceae (Burge & Manchester, 2008). Fossil records of *Paliurus*, the closest family to *Ziziphus* within Rhamnaceae based on molecular phylogenetic and morphological analysis, were numerously explored in North America, Europe, and Asia (Han et al., 2016; Islam & Simmons, 2006). The earliest identical fruit fossil record of *Paliurus* in Eastern Asia dated back to about the middle to late Eocene (Han et al., 2016). Although it cannot precisely determine the divergence time of these two closely related genera, the appearance of the diagnostic characteristics of modern *Paliurus* implied that species of *Ziziphus* were relatively old. Based on genomic sequences of species from Rosales, Liu et al. (2014) found that *Z. jujuba* diverged from other Rosales species at about 79.9 Ma. It was revealed that the two intraspecific groups of sour jujube diverged at about 55.86 Ma. The initial desertification in the Asian interior is thought to be one of the most prominent climate changes in the Northern Hemisphere during the Cenozoic era (An et al., 2001; Guo et al., 2002; Ruddiman & Kutzbach, 1989). Regional tectonic changes and ongoing global climate oscillations were probable causes of the intraspecific divergence revealed in sour jujube. Detailed information about this may need further investigation with more data, such as genomic SNPs.

The uplift of mountain regions may provide suitable habitats for plant species to survive during the Quaternary glaciation. It has been widely accepted that the Zhongtiao Mountains locating in areas between the Loess Plateau and Qinling Mountains in Central China harbor relatively rich vegetation diversity and serve as main glacial refugia for temperate plant species in the Quaternary glaciation, such as *Acer mono* Thunb. ex Murray (Liu, 2013) and *X. sorbifolium* (Zhu, 2016), resulting from appropriate temperature and adequate precipitation (Wang et al., 2004). In Maxent analysis, it was revealed that the higher suitable distribution area of sour jujube in China located at the Loess Plateau in different periods. During field sampling, it was found that sour jujube was distributed continuously in this area. Furthermore, nucleotide variation of central populations distributed in the Loess Plateau was much higher than that of the marginal populations (Table S8). Network analysis found that the chloroplast haplotype located in the Loess Plateau was more ancient. Combining these findings, we can speculate that a potential glacial refuge for sour jujube may locate in areas between the Loess Plateau and the Qinling Mountains, probably in areas adjacent to the Zhongtiao Mountains. Another higher suitable distribution area of sour jujube in LGM is located at areas adjacent to the Tianmu Mountains, which was also thought to serve as the main glacial refuge for temperate plant species in China, such as *Ostryopsis davidiana* Decne. (Tian et al., 2009), *Juglans mandshurica* Maxim. (Bai et al., 2018) and *Ginkgo biloba* L. (Zhao et al., 2019). However, in the present we did not collect samples from this area, maybe because the environmental conditions were not suitable for sour jujube, such as high moisture. These mountainous areas were important for the glacial survival of sour jujube and for preserving most of the extant nucleotide variation. This provides additional evidence for the in situ survival of plant species in Central China during the Quaternary glaciation and local expansion during interglacial or postglacial, which was similar to the scenarios revealed in other regions (Wang, Abbott, et al., 2010; Wang, Ikeda, et al., 2010). The most important refugia for sour jujube appear to have been located at the Loess Plateau in Central China and areas adjacent to the Tianmu Mountains in East China, which provides new insights for phylogeographic research of temperate plant species distributed in Central China. Furthermore, samples from other sour jujube distribution areas, such as Inner Mongolia Autonomous Region and Liaoning Province were not collected in the present study, but based on the above analysis, we may speculate that the populations distributed in these areas may also migrate or colonize from the potential refugia or central populations revealed in the present study. Future research may include samples from these areas to explicitly test our speculation.

## 5 CONCLUSION

This study provided new insights into the genetic diversity, population structure, and demography of *Z. jujuba* var. *spinosa*. The genetic diversity of sour jujube was relatively high. Results of AMOVA showed that most of the total variation was attributed to variation within populations and high genetic differentiation among populations was detected. Geographic distance and environmental difference contributed to the genetic differentiation among populations. Structure analysis successfully uncovered two intraspecific genetic groups, central population and marginal population corresponding to their geographic locations. Population demographic history of sour jujube illustrated a contraction‐expansion model responding to the Quaternary climate oscillations. Under future environmental conditions, the distribution habitat of sour jujube may shift to high altitudinal areas. Two potential glacial refugia were uncovered. We are confident that the information provided in the present study will be very helpful to the sour jujube management, conservation, and breeding activities.

## AUTHOR CONTRIBUTIONS

**Shuhui Du:** Formal analysis (equal); writing – original draft (lead). **Xiaoyan Hu:** Formal analysis (equal); writing – original draft (supporting). **Wendong Yu:** Investigation (equal). **Zhaoshan Wang:** Investigation (equal).

## CONFLICT OF INTEREST

The authors declare that the research was conducted in the absence of any commercial or financial relationships that could be construed as a potential conflict of interest.

## Supporting information


Appendix S1.

## Data Availability

The data presented in this study are openly available in NCBI GenBank with accession number MW371295‐MW372934 for single‐copy nuclear gene markers and NCBI SRA with accession number ON611607‐ON611627 for chloroplast genome data.

## References

[ece39101-bib-0001] Abbott, R. J. , & Comes, H. P. (2004). Evolution in the Arctic: A phylogeographic analysis of the circumarctic plant, *Saxifraga oppositifolia* (purple saxifrage). New Phytologist, 161, 211–224.

[ece39101-bib-0002] Aiello‐Lammens, M. E. , Boria, R. A. , Radosavljevic, A. , Vilela, B. , & Anderson, R. P. (2015). spThin: An R package for spatial thinning of species occurrence records for use in ecological niche models. Ecography, 5, 541–545.

[ece39101-bib-0003] An, Z. S. , John, E. K. , Warren, L. P. , & Stephen, C. P. (2001). Evolution of Asian monsoons and phased uplift of the Himalaya–Tibetan plateau since late Miocene times. Nature, 411, 62–66. 11333976 10.1038/35075035

[ece39101-bib-0004] Bai, W. N. , Liao, W. J. , & Zhang, D. Y. (2010). Nuclear and chloroplast DNA phylogeography reveal two refuge areas with asymmetrical gene flow in a temperate walnut tree from East Asia. New Phytologist, 188, 892–901. 20723077 10.1111/j.1469-8137.2010.03407.x

[ece39101-bib-0005] Bai, W. N. , Yan, P. C. , Zhang, B. W. , Woeste, K. E. , & Zhang, D. Y. (2018). Demographically idiosyncratic responses to climate change and rapid Pleistocene diversification of the walnut genus *Juglans* (Juglandaceae) revealed by whole‐genome sequences. New Phytologist, 4, 1726–1736. 10.1111/nph.14917 29178135

[ece39101-bib-0006] Beerli, P. (2001). Maximum likelihood estimation of a migration matrix and effective population sizes in n subpopulations by using a coalescent approach. Proceedings of the National Academy of Sciences of the United States of America, 8, 4563–4568. 10.1073/pnas.081068098 PMC31874 11287657

[ece39101-bib-0007] Burge, D. O. , & Manchester, S. R. (2008). Fruit morphology, fossil history, and biogeography of *Paliurus* (Rhamnaceae). International Journal of Plant Sciences, 169, 1066–1085.

[ece39101-bib-0008] Campana, M. , Hunt, H. , Jones, H. , & White, J. (2011). CorrSieve: Software for summarizing and evaluating structure output. Molecular Ecology Resources, 11, 349–352. 21429142 10.1111/j.1755-0998.2010.02917.x

[ece39101-bib-0009] Charlesworth, D. (2003). Effects of inbreeding on the genetic diversity of populations. Philosophical Transactions of the Royal Society B Biological Sciences, 358, 1051. 10.1098/rstb.2003.1296 PMC1693193 12831472

[ece39101-bib-0010] Du, F. K. , Meng, H. , Wang, W. , Mao, K. , & Hampe, A. (2017). Phylogeography of *Quercus aquifolioides* provides novel insights into the Neogene history of a major global hotspot of plant diversity in south‐West China. Journal of Biogeography, 44, 294–307.

[ece39101-bib-0011] Du, S. H. , Wang, Z. S. , Ingvarsson, P. K. , Wang, D. S. , Wang, J. H. , Wu, Z. Q. , Tembrock, L. R. , & Zhang, J. G. (2015). Multilocus analysis of nucleotide variation and speciation in three closely related *Populus* (Salicaceae) species. Molecular Ecology, 24, 4994–5005. 26334549 10.1111/mec.13368

[ece39101-bib-0012] Du, S. H. , Ye, Z. Y. , Hu, X. Y. , Liu, S. Y. , & Wang, Z. S. (2020). Phylogeographic investigation of *Elaeagnus mollis* revealed potential glacial refugia and allopatric divergence in Central China. Plant Systematics and Evolution, 306, 68.

[ece39101-bib-0013] Duan, N. , Bai, Y. , Sun, H. , Wang, N. , Ma, Y. , Li, M. , Wang, X. , Jiao, C. , Legall, N. , Mao, L. , & Wan, S. (2017). Genome re‐sequencing reveals the history of apple and supports a two‐stage model for fruit enlargement. Nature Communications, 8, 249. 10.1038/s41467-017-00336-7 PMC5557836 28811498

[ece39101-bib-0014] Emerson, K. J. , Merz, C. R. , Catchen, J. M. , Hohenlohe, P. A. , Cresko, W. A. , Bradshaw, W. E. , & Holzapfel, C. M. (2010). Resolving postglacial phylogeography using high‐throughput sequencing. Proceedings of the National Academy of Sciences of the United States of America, 107, 16196–16200. 20798348 10.1073/pnas.1006538107 PMC2941283

[ece39101-bib-0015] Evanno, G. S. , Regnaut, S. J. , & Goudet, J. (2005). Detecting the number of clusters of individuals using the software STRUCTURE: A simulation study. Molecular Ecology, 14, 2611–2620. 15969739 10.1111/j.1365-294X.2005.02553.x

[ece39101-bib-0016] Excoffier, L. , Laval, G. , & Schneider, S. (2005). Arlequin (version 3.0): An integrated software package for population genetics data analysis. Evolutionary Bioinformatics Online, 1, 47–50. PMC2658868 19325852

[ece39101-bib-0017] Falush, D. , Stephens, M. , & Pritchard, J. K. (2003). Inference of population structure using multilocus genotype data: Linked loci and correlated allele frequencies. Genetics, 164, 1567–1587. 12930761 10.1093/genetics/164.4.1567 PMC1462648

[ece39101-bib-0018] Felsenstein, J. (1988). Phylogenies from molecular sequences: Inference and reliability. Annual Review of Genetics, 22, 521–565. 10.1146/annurev.ge.22.120188.002513 3071258

[ece39101-bib-0019] Feng, X. , Zhou, H. , Zulfiqar, S. , Luo, X. , Hu, Y. , Feng, L. , Malvolti, M. E. , Woeste, K. , & Zhao, P. (2018). The phytogeographic history of common walnut in China. Frontiers in Plant Science, 9, 1399. 30298084 10.3389/fpls.2018.01399 PMC6160591

[ece39101-bib-0020] French, C. M. , Deutsch, M. S. , Chávez, G. , Almora, C. E. , & Brown, J. L. (2019). Speciation with introgression: Phylogeography and systematics of the *Ameerega petersi* group (Dendrobatidae). Molecular Phylogenetics and Evolution, 138, 31–42. 31125660 10.1016/j.ympev.2019.05.021

[ece39101-bib-0021] Gao, J. D. , Yang, T. Z. , Wang, Q. , Wu, Y. X. , & Tian, J. B. (2008). Situation and progress suggestions on development of sour jujube in Shanxi. In W. Li (Ed.), National symposium on Research and Development of fruit tree planting resources. China Agricultural Express.

[ece39101-bib-0022] Gao, Y. D. , Gao, X. F. , & Harris, A. (2019). Species boundaries and parapatric speciation in the complex of alpine shrubs, *Rosa sericea* (Rosaceae), based on population genetics and ecological tolerances. Frontiers in Plant Science, 10, 321. 30936888 10.3389/fpls.2019.00321 PMC6432857

[ece39101-bib-0023] Gonzales, E. , Hamrick, J. L. , & Chang, S. M. (2008). Identification of glacial refugia in South‐Eastern North America by phylogeographical analyses of a forest understorey plant, *Trillium cuneatum* . Journal of Biogeography, 35, 844–852.

[ece39101-bib-0024] Guo, M. X. , Zhang, Z. R. , Li, S. P. , Lian, Q. , Fu, P. C. , He, Y. L. , Qiao, J. X. , Xu, K. K. , Liu, L. P. , Wu, M. Y. , Du, Z. R. , Li, S. N. , Wang, J. J. , Shao, P. Y. , Yu, Q. , Xu, G. , Li, D. K. , Wang, Y. K. , Tian, S. , … Zhao, X. S. (2021). Genomic analyses of diverse wild and cultivated accessions provide insights into the evolutionary history of jujube. Plant Biotechonlog Journal, 19, 517–531. 10.1111/pbi.13480 PMC7955879 32946650

[ece39101-bib-0025] Guo, Z. T. , Ruddiman, W. F. , Hao, Q. Z. , Wu, H. B. , Qiao, Y. S. , Zhu, R. X. , Peng, S. Z. , Wei, J. J. , Yuan, B. Y. , & Liu, T. S. (2002). Onset of Asian desertification by 22 Myr ago inferred from loess deposits in China. Nature, 416, 159–163. 11894089 10.1038/416159a

[ece39101-bib-0026] Hall, T. A. (1999). BioEdit: A user‐friendly biological sequence alignment editor and analysis program for windows 95/98/NT. In Nucleic acids symposium series (Vol. 41, pp. 95–98). Information Retrieval Ltd.

[ece39101-bib-0027] Han, M. , Chen, G. , Shi, X. G. , & Jin, J. H. (2016). Earliest fossil fruit record of the genus *Paliurus* (Rhamnaceae) in eastern Asia. Science China Earth Sciences, 59, 824–830.

[ece39101-bib-0028] Hazzouri, K. M. , Flowers, J. M. , Visser, H. J. , Khierallah, H. S. , Rosas, U. , Pham, G. M. , Meyer, R. S. , Johansen, C. K. , Fresquez, Z. A. , Masmoudi, K. , & Haider, N. (2015). Whole genome re‐sequencing of date palms yields insights into diversification of a fruit tree crop. Nature Communications, 6, 8824. 10.1038/ncomms9824 PMC4667612 26549859

[ece39101-bib-0029] Hewitt, G. (2000). The genetic legacy of the quaternary ice ages. Nature, 405, 907–913. 10879524 10.1038/35016000

[ece39101-bib-0030] Hewitt, G. M. (2004). Genetic consequences of climatic oscillations in the quaternary. Philosophical Transactions of the Royal Society of London Series B‐Biological Sciences, 359, 183–195. 15101575 10.1098/rstb.2003.1388 PMC1693318

[ece39101-bib-0031] Hou, Z. , Wang, Z. S. , Ye, Z. Y. , Du, S. H. , Liu, S. Y. , & Zhang, J. G. (2018). Phylogeographic analyses of a widely distributed *Populus davidiana*: Further evidence for the existence of glacial refugia of cool‐temperate deciduous trees in northern East Asia. Ecology and Evolution, 8, 13014–13026. 30619601 10.1002/ece3.4755 PMC6308874

[ece39101-bib-0032] Hu, X. Y. , Du, S. H. , Wang, Z. S. , & Han, Y. Z. (2021). Genetic diversity and genetic structure of sour jujube in Shanxi. Forestry Research, 33, 137–144.

[ece39101-bib-0033] Hu, X. Y. , Du, S. H. , & Han, Y. Z. (2021). A genome‐scale mining of single‐copy nuclear gene markers for *Ziziphus jujuba* var. *spinosa* and implications for genetic study. Pakistan Journal of Botany, 4, 1253–1258.

[ece39101-bib-0034] Huang, J. , Zhang, C. M. , Zhao, X. , Fei, Z. J. , Wan, K. K. , Zhang, Z. , Pang, X. M. , Yin, X. , Bai, Y. , Sun, X. Q. , Gao, L. Z. , Li, R. Q. , Zhang, J. B. , & Li, X. G. (2016). The Jujube genome provides insights into genome evolution and the domestication of sweetness/Acidity taste in fruit trees. Plos Genetics, 12, e1006433. 28005948 10.1371/journal.pgen.1006433 PMC5179053

[ece39101-bib-0035] Hudson, R. R. , Kreitman, M. , & Aguadé, M. (1987). A test of neutral molecular evolution based on nucleotide data. Genetics, 116, 153–159. 3110004 10.1093/genetics/116.1.153 PMC1203113

[ece39101-bib-0036] Islam, M. B. , & Simmons, M. P. (2006). A thorny dilemma: Testing alternative intrageneric classifications within *Ziziphus* (Rhamnaceae). Systematic Botany, 4, 826–842.

[ece39101-bib-0037] Ji, L. T. , Zheng, T. Y. , Chen, Q. , Zhong, J. J. , & Kang, B. (2020). Responses of potential suitable area of *Paris verticillata* to climate change and its dominant climate factors. Chinese Journal of Applied Ecology, 31, 81–96. 10.13287/j.1001-9332.202001.012 31957384

[ece39101-bib-0038] Kang, D. D. , Han, L. H. , Ma, P. F. , Wei, X. Z. , & Bi, R. C. (2008). Comparison on characters of leaf anatomy of *Ziziphus jujuba* var. *spinosain* different geography environment. Scientia Silvae Sinicae, 44, 135–139.

[ece39101-bib-0039] Khan, G. , Zhang, F. , Gao, Q. , Fu, P. , Yu, Z. , & Chen, S. (2018). Spiroides shrubs on Qinghai‐Tibetan plateau: Multilocus phylogeography and palaeodistributional reconstruction of *Spiraea alpina* and *S. mongolica* (Rosaceae). Molecular Phylogenetics & Evolution, 123, 137–148. 29462675 10.1016/j.ympev.2018.02.009

[ece39101-bib-0040] Lafontaine, G. D. , Turgeon, J. , & Payette, S. (2010). Phylogeography of white spruce (*Picea glauca*) in eastern North America reveals contrasting ecological trajectories. Journal of Biogeography, 37, 741–751.

[ece39101-bib-0041] Lange, J. D. , Bastide, H. , Lack, J. B. , & Pool, J. E. (2021). A population genomic assessment of three decades of evolution in a natural *drosophila* population. Molecualr Biology and Evolution, 39, msab368. 10.1093/molbev/msab368 PMC8826484 34971382

[ece39101-bib-0042] Leigh, J. W. , & Bryant, D. (2015). PopART: Full‐feature software for haplotype network construction. Methods in Ecology and Evolution, 9, 1110–1116.

[ece39101-bib-0043] Li, H. , & Fu, X. (1993). Statistical tests of neutrality of mutations. Genetics, 133, 693–709. 8454210 10.1093/genetics/133.3.693 PMC1205353

[ece39101-bib-0044] Li, Y. , Cao, K. , Zhu, G. , Fang, W. , Chen, C. , Wang, X. , Zhao, P. , Guo, J. , Ding, T. , Guan, L. , & Zhang, Q. (2019). Genomic analyses of an extensive collection of wild and cultivated accessions provide new insights into peach breeding history. Genome Biology, 20, 36. 30791928 10.1186/s13059-019-1648-9 PMC6383288

[ece39101-bib-0045] Li, Z. , Ren, S. , Chang, Z. R. , Yan, H. X. , Chen, Y. G. , & Fu, X. T. (2017). Content comparison of three components in Ziziphi Spinosae semen pieces. Chinese Journal of New Drugs, 1, 91–96.

[ece39101-bib-0046] Librado, P. , & Rozas, J. (2009). DnaSP v5: A software for comprehensive analysis of DNA polymorphism data. Bioinformatics, 25, 1451–1452. 19346325 10.1093/bioinformatics/btp187

[ece39101-bib-0047] Liu, C. P. (2013). Study on genetic structure and phylogeography of Acer mono natural populations. Northeast Forestry University.

[ece39101-bib-0048] Liu, M. J. (1993). Advances in taxonomy study on the genus Ziziphus in China. Beijing Medical University, Beijing University.

[ece39101-bib-0049] Liu, M. J. , Zhao, J. , Cai, Q. L. , Liu, G. C. , Wang, J. R. , Zhao, Z. H. , Liu, P. , Dai, L. , Yan, G. , & Wang, W. J. (2014). The complex jujube genome provides insights into fruit tree biology. Nature Communications, 5, 5315. 10.1038/ncomms6315 PMC4220462 25350882

[ece39101-bib-0050] Liu, X. , Wang, Z. S. , Shao, W. H. , Ye, Z. Y. , & Zhang, J. G. (2016). Phylogenetic and taxonomic status analyses of the *Abaso* section from multiple nuclear genes and plastid fragments reveal new insights into the North America origin of *Populus* (Salicaceae). Frontiers in Plant Science, 7, e266. 10.3389/fpls.2016.02022 PMC5209371 28101098

[ece39101-bib-0051] Manni, F. , Guérard, E. , & Heyer, E. (2004). Geographic patterns of (genetic, morphologic, linguistic) variation: How barriers can be detected by using Monmonier's algorithm. Human Biology, 76, 173–190. 15359530 10.1353/hub.2004.0034

[ece39101-bib-0052] Mantel, N. (1967). The detection of disease clustering and a generalized regression approach. Cancer Research, 27, 209–220. 6018555

[ece39101-bib-0053] Mu, X. Y. , Tong, L. , Sun, M. , Zhu, Y. X. , Wen, J. , Lin, Q. W. , & Liu, B. (2020). Phylogeny and divergence time estimation of the walnut family (Juglandaceae) based on nuclear RAD‐seq and chloroplast genome data. Molecular Phylogenetics and Evolution, 147, 106802. 32217170 10.1016/j.ympev.2020.106802

[ece39101-bib-0054] Nei, M. (1987). Molecular evolutionary genetics. Science, 235, 599. 17758251 10.1126/science.235.4788.599

[ece39101-bib-0055] Paola, P. , Keith, W. , Francesca, C. , Stefano, D. L. , Marco, C. , Irene, O. , Virginia, T. , Jo, C. , Hemery, G. E. , & Sergio, M. (2017). Rethinking the history of common walnut (*Juglans regia* L.) in Europe: Its origins and human interactions. PLoS One, 12, e0172541. 28257470 10.1371/journal.pone.0172541 PMC5336217

[ece39101-bib-0056] Peng, J. Y. (1991). Pollen mrophology of jujube and sour jujube‐taxonomic classification and phylogeny of jujube cultivation. Heibei Agriculture University.

[ece39101-bib-0057] Phillips, S. J. , Anderson, R. P. , & Schapire, R. E. (2006). Maximum entropy modeling of species geographic distributions. Ecological Modelling, 190, 231–259.

[ece39101-bib-0058] Phillips, S. J. , & Dudík, M. (2008). Modeling of species distributions with Maxent: New extensions and a comprehensive evaluation. Ecography, 31, 161–175.

[ece39101-bib-0059] Posada, D. (2008). ModelTest: Phylogenetic model averaging. Molecular Biology and Evolution, 25, 1253–1256. 18397919 10.1093/molbev/msn083

[ece39101-bib-0060] Qian, H. , & Ricklefs, R. E. (2001). Palaeovegetation (communications arising): Diversity of temperate plants in East Asia. Nature, 6852, 130–131. 10.1038/35093166 11557970

[ece39101-bib-0061] Qiu, Y. X. , Fu, C. X. , & Comes, H. P. (2011). Plant molecular phylogeography in China and adjacent regions: Tracing the genetic imprints of quaternary climate and environmental change in the world's most diverse temperate flora. Molecular Phylogenetics and Evolution, 1, 225–244. 10.1016/j.ympev.2011.01.012 21292014

[ece39101-bib-0062] Qu, Z. Z. (1982). History, type and application of sour jujube. Journal of Hebei Agriculture University, 12, 112–117.

[ece39101-bib-0063] Ramos‐Onsins, S. E. , & Rozas, J. (2002). Statistical properties of new neutrality tests against population growth. Molecular Biology and Evolution, 19, 2092–2100. 12446801 10.1093/oxfordjournals.molbev.a004034

[ece39101-bib-0064] Ramos‐Onsins, S. E. , Puerma, E. , Bala‐Alcaide, D. , Salguero, D. , & Aguad, M. (2010). Multilocus analysis of variation using a large empirical data set: Phenylpropanoid pathway genes in *Arabidopsis thaliana* . Molecular Ecology, 17, 1211–1223. 10.1111/j.1365-294X.2007.03633.x 18221273

[ece39101-bib-0065] Ren, G. , Mateo, R. G. , Liu, J. , Suchan, T. , Alvarez, N. , Guisan, A. , Conti, E. , & Salamin, N. (2017). Genetic consequences of quaternary climatic oscillations in the Himalayas: *Primula tibetica* as a case study based on restriction site‐associated DNA sequencing. New Phytologist, 213, 1500–1512. 27696413 10.1111/nph.14221

[ece39101-bib-0066] Riddle, A. B. (2010). Phylogeography: Retrospect and prospect. Journal of Biogeography, 36, 3–15.

[ece39101-bib-0067] Rosenberg, N. A. (2004). DISTRUCT: A program for the graphical display of population structure. Molecular Ecology Notes, 4, 137–138.

[ece39101-bib-0068] Ruddiman, W. F. , & Kutzbach, J. E. (1989). Forcing of late Cenozoic northern hemisphere climate by plateau uplift in southern Asia and the American west. Journal of Geophysical Research, 15, 18409–18427.

[ece39101-bib-0069] Savolainen, O. , Pyhjrvi, T. , & Knürr, T. (2007). Gene flow and local adaptation in trees. Annual Review of Ecology Evolution & Systematics, 38, 595–619.

[ece39101-bib-0070] Shao, F. X. , Wang, S. , Chen, J. H. , Hong, R. Y. , Chen, J. , & Wang, J. (2020). Stamen morphological development and pollen viability of *Ziziphus jujuba* ‘Zhongqiusucui’. Plant Physiology Journal, 56, 16–31.

[ece39101-bib-0071] Shen, L. Y. , Luo, H. , Wang, X. L. , Wang, X. M. , Qiu, X. J. , Liu, H. , Zhou, S. S. , Jia, K. H. , Nie, S. , Bao, Y. T. , Zhang, R. G. , Yun, Q. Z. , Chai, Y. H. , Lu, J. Y. , Li, Y. , Zhao, S. W. , Mao, J. F. , Jia, S. G. , & Mao, Y. M. (2021). Chromosome‐scale genome assembly for Chinese sour jujube and insights into its genome evolution and domestication signature. Frontiers in Plant Science, 12, 773090. 34899800 10.3389/fpls.2021.773090 PMC8652243

[ece39101-bib-0072] Simpson, G. , Solymos, P. , Stevens, M. H. H. , & Wagner, H. (2010). Vegan: Community ecology package. R package version, 117‐114.

[ece39101-bib-0073] Suchard, M. A. , Philippe, L. , Guy, B. , Ayres, D. L. , Drummond, A. J. , & Andrew, R. (2018). Bayesian phylogenetic and phylodynamic data integration using BEAST 1.10. Virus Evolution, 4, vey016. 29942656 10.1093/ve/vey016 PMC6007674

[ece39101-bib-0074] Tajima, F. (1989). Statistical method for testing the neutral mutation hypothesis by DNA polymorphism. Genetics, 123, 585–595. 2513255 10.1093/genetics/123.3.585 PMC1203831

[ece39101-bib-0075] Tamura, K. , Stecher, G. , Peterson, D. , Filipski, A. , & Kumar, S. (2013). MEGA6: Molecular evolutionary genetics analysis version 6.0. Molecular Biology and Evolution, 12, 2725–2729. 10.1093/molbev/mst197 PMC3840312 24132122

[ece39101-bib-0076] Thompson, J. (1997). The CLUSTAL_X windows interface: Flexible strategies for multiple sequence alignment aided by quality analysis tools. Nucleic Acids Research, 25, 4876–4882. 9396791 10.1093/nar/25.24.4876 PMC147148

[ece39101-bib-0077] Tian, B. , Liu, R. , Wang, L. , Qiu, Q. , Chen, K. , & Liu, J. (2009). Phylogeographic analyses suggest that a deciduous species (*Ostryopsis davidiana* Decne., Betulaceae) survived in northern China during the last glacial maximum. Journal of Biogeography, 36, 2148–2155.

[ece39101-bib-0078] Walther, G. R. , Berger, S. , & Sykes, M. T. (2005). An ecological 'footprint' of climate change. Proceedings of the Royal Society B: Biological Sciences, 272, 1427–1432. 10.1098/rspb.2005.3119 PMC1559830 16011916

[ece39101-bib-0079] Wang, D. S. , Wang, Z. S. , Kang, X. Y. , & Zhang, J. G. (2019). Genetic analysis of admixture and hybrid patterns of *Populus hopeiensis* and *P. tomentosa* . Scientific Reprots, 1, 4821. 10.1038/s41598-019-41320-z PMC6423230 30886279

[ece39101-bib-0080] Wang, H. , Kang, M. , Liu, W. , & Liu, Q. (2004). Advance in studies on flora and vegetation in Zhongtiao Mountain. Journal of Beijing Normal University (Natural Science), 40, 676–683.

[ece39101-bib-0081] Wang, L. Y. , Abbott, R. J. , Zheng, W. , Chen, P. , Wang, Y. , & Liu, J. (2010). History and evolution of alpine plants endemic to the Qinghai‐Tibetan plateau: *Aconitum gymnandrum* (Ranunculaceae). Molecular Ecology, 18, 709–721. 10.1111/j.1365-294X.2008.04055.x 19175501

[ece39101-bib-0082] Wang, L. Y. , Ikeda, H. , Liu, T. L. , Wang, Y. J. , & Liu, J. Q. (2010). Repeated range expansion and glacial endurance of *Potentilla glabra* (Rosaceae) in the Qinghai‐Tibetan plateau. Journal of Integrative Plant Biology, 51, 698–706. 10.1111/j.1744-7909.2009.00818.x 19566648

[ece39101-bib-0083] Wang, Q. , Abbott, R. J. , Yu, Q. S. , Lin, K. , & Liu, J. Q. (2013). Pleistocene climate change and the origin of two desert plant species, *Pugionium cornutum* and *Pugionium dolabratum* (Brassicaceae), in Northwest China. New Phytologist, 1, 277–287. 10.1111/nph.12241 23550542

[ece39101-bib-0084] Wang, Z. Q. , Wu, C. Y. , Yang, Z. , Yang, F. , & Wu, Y. C. (2018). Effect of saline‐alkali stress on growth, physiological and biochemical characteristics of wild jujube seedlings. Agricultural Research in the Arid Areas, 36, 153–160.

[ece39101-bib-0085] Watterson, G. A. (1975). On the number of segregating sites in genetical models without recombination. Theoretical Population Biology, 7, 256–276. 1145509 10.1016/0040-5809(75)90020-9

[ece39101-bib-0086] Wen, Y. X. , Gan, H. M. , Shi, S. Q. , Jiang, Z. P. , Wu, L. L. , & Chu, J. M. (2020). Phylogeography of *Tamarix austromongolica* based on the sequences of chloroplast and nuclear gene fragments. Scientia Silvae Sinicae, 56, 55–64.

[ece39101-bib-0087] Wright, S. , & Maxson, L. E. R. (1968). Evolution and the genetics of populations. Physiological & Biochemical Zoology, 8, 1191–1192.

[ece39101-bib-0088] Wright, S. I. , & Gaut, B. S. (2005). Molecular population genetics and the search for adaptive evolution in plants. Molecular Biology and Evolution, 22, 506–519. 15525701 10.1093/molbev/msi035

[ece39101-bib-0089] Wu, J. , Wang, Y. , Xu, J. , Korban, S. S. , Fei, Z. , Tao, S. , Ming, R. , Tai, S. , Khan, A. M. , Postman, J. D. , & Gu, C. (2018). Diversification and independent domestication of Asian and European pears. Genome Biology, 19, 77. 29890997 10.1186/s13059-018-1452-y PMC5996476

[ece39101-bib-0090] Wu, Z. (1982). Flora of China. Science Press.

[ece39101-bib-0091] Yan, Y. , Li, Q. , Du, C. H. , Jia, J. P. , Feng, H. X. , & Qin, X. M. (2017). Investigation of the potentially effective components of semen Ziziphi Spinosae based on “in vitro to in vivo” translation approach. Acta Pharmaceutica Sinica, 2, 283–290. 29979520

[ece39101-bib-0092] Yu, G. , Chen, X. , Ni, J. , Cheddadi, R. , Guiot, J. , Han, H. , Harrison, S. P. , Huang, C. , Ke, M. , & Kong, Z. (2000). Palaeovegetation of China: A pollen data‐based synthesis for the mid‐Holocene and last glacial maximum. Journal of Biogeography, 27, 635–664.

[ece39101-bib-0093] Zeng, Y. F. , Wang, W. T. , Liao, W. J. , Wang, H. F. , & Zhang, D. Y. (2016). Multiple glacial refugia for cool‐temperate deciduous trees in northern East Asia: The Mongolian oak as a case study. Molecular Ecology, 24, 5676–5691. 10.1111/mec.13408 26439083

[ece39101-bib-0094] Zhang, C. M. (2013). Development of SSR primers and genetic diversity of sour jujube. Northwest A&F University.

[ece39101-bib-0095] Zhang, C. M. , Huang, J. , Xiao, Y. , Lian, C. , & Li, X. G. (2015). Genetic diversity and population structure of sour jujube, *Ziziphus acidojujuba* . Tree Genetics & Genomes, 11, 809.

[ece39101-bib-0096] Zhang, C. M. , Yin, X. , Li, X. G. , Huang, J. , Wang, C. Z. , & Lian, C. L. (2013). Genetic diversity of sour jujube along the Yellow River. Journal of Northwest A&F University‐Natural Science Edition, 41, 107–119.

[ece39101-bib-0097] Zhang, J. Y. , Mao, X. H. , & Zhang, Y. Y. (2014). Analysis on genetic diversity in *Ziziphus acidojujuba* resources based on RAMP markers. Nonwood Forest Research, 2, 14–18.

[ece39101-bib-0098] Zhao, G. H. , Cui, X. Y. , Sun, J. J. , Li, T. T. , Wang, Q. , Ye, X. Z. , & Fan, B. G. (2021). Analysis of the distribution pattern of Chinese Ziziphus jujuba under climate change based on optimized biomod2 and MaxEnt models. Ecological Indicators, 132, 108256.

[ece39101-bib-0099] Zhao, Y. P. , Fan, G. , Yin, P. P. , Sun, S. , & Ge, S. (2019). Resequencing 545 ginkgo genomes across the world reveals the evolutionary history of the living fossil. Nature Communications, 10, 1–10. 10.1038/s41467-019-12133-5 PMC6744486 31519986

[ece39101-bib-0100] Zhao, Z. , & Piperno, D. R. (2000). Late Pleistocene/Holocene environments in the middle Yangtze River valley, China and rice (*Oryza sativa* L.) domestication: The phytolith evidence. Geoarchaeology, 15, 203–222.

[ece39101-bib-0101] Zhao, Z. X. (2016). Evalution on the slected sour jujube seedings resistant to salt. Shihezi University.

[ece39101-bib-0102] Zheng, H. , Fan, L. , Milne, R. I. , Zhang, L. , Wang, Y. , & Mao, K. (2017). Species delimitation and lineage separation history of a species complex of aspens in China. Frontiers in Plant Science, 8, 375. 28377782 10.3389/fpls.2017.00375 PMC5359289

[ece39101-bib-0103] Zheng, X. , & Ge, S. (2010). Ecological divergence in the presence of gene flow in two closely related *Oryza* species (*Oryza rufipogon* and *O. nivara*). Molecular Ecology, 19, 2439–2454. 20653085 10.1111/j.1365-294x.2010.04674.x

[ece39101-bib-0104] Zheng, Z. , Yuan, B. Y. , & Petit‐Maire, N. (1998). Paleoenvironments in China during the last glacial maximum and the Holocene optimum. Episodes, 21, 152–158.

[ece39101-bib-0105] Zhu, R. B. (2016). Phylogeography of Xanthoceras Sorbifolium Bunge, an endemic plant to China. Northwest A&F University.

