# Supplementary material for: Genetic diversity and population dynamic of Ziziphus jujuba var. spinosa (Bunge) Hu ex H. F. Chow in Central China
Source: Ecol Evol. 2022 Jul 24;12(7):e9101. doi: 10.1002/ece3.9101 (PMC9309028; doi:10.1002/ece3.9101)
Supplement: Supplementary file 1 — Appendix S1. [file ECE3-12-e9101-s001.docx]

Appendix

Table S1 Sampled populations of *Z. jujuba* var. *spinosa* in this study

| Number | Province | Population | Population code | Geographical information | No. of samples |
| --- | --- | --- | --- | --- | --- |
| 1 | Hebei | Longhua County, Chengde | HBCD | 41.39°N 117.85°E | 19 |
| 2 |  | Haigang District,Qinhuangdao | HBQHD | 40.01°N 119.52°E | 14 |
| 3 |  | Renze District, Xingtai | HBXT | 37.13°N 114.76°E | 19 |
| 4 | Shanxi | Changzhi County，Changzhi | CZCZ | 35.35°N 112.56°E | 20 |
| 5 |  | Yungang District，Datong | DTYG | 39.61°N 112.86°E | 18 |
| 6 |  | Taigu District，Jinzhong | JZTG | 36.91°N 112.42°E | 19 |
| 7 |  | Xiaoyi County，Lvliang | LLXY | 36.65°N 111.78°E | 20 |
| 8 |  | Ruicheng County，Yuncheng | YCRC | 34.69°N 110.69°E | 19 |
| 9 | Shandong | Laoshan District, Qingdao | SDLS | 36.18°N 120.54°E | 16 |
| 10 |  | Mengyin County, Linyi | LYMY | 35.56°N 118.34°E | 18 |
| 11 |  | Taishan District, Taian | SDTA | 36.21°N 117.12°E | 15 |
| 12 | Tianjin | Wuqing District, Tianjin | TJWQ | 39.48°N 117.02°E | 9 |
| 13 | Ningxia | Yongning County, Yinchuan | NXYC | 38.18°N 106.19°E | 15 |
| 14 | Shaanxi | Nanzheng County, Hanzhong | SXHZ | 32.92°N 109.79°E | 12 |
| 15 |  | Linwei District, Weinan | SXWN | 34.39°N 113.44°E | 11 |
| 16 | Henan | Dengzhou County, Nanyang | HNNY | 32.64°N 111.93°E | 10 |
| 17 |  | Lushi County, Samenxia | HNSMX | 34.08°N 110.94°E | 12 |
| 18 |  | Yiyang County, Luoyang | HNLY | 34.49°N 112.13°E | 15 |
| 19 |  | Zhongmou County,Zhengzhou | HNZZ | 34.88°N 114.03°E | 13 |
| 20 | Anhui | Shucheng District, Liuan | AHLA | 31.41°N 117.08°E | 14 |
| 21 | Hubei | Nanzhang County, Xiangyang | HBXY | 31.71°N 111.78°E | 20 |

Table S2 Correlation analysis of 23 environmental variables

|  | bio1 | bio2 | bio3 | bio4 | bio5 | bio6 | bio7 | bio8 | bio9 | bio10 | bio11 | bio12 | bio13 | bio14 | bio15 | bio16 | bio17 | bio18 | bio19 | elevation | slope | aspect | soil |
| --- | --- | --- | --- | --- | --- | --- | --- | --- | --- | --- | --- | --- | --- | --- | --- | --- | --- | --- | --- | --- | --- | --- | --- |
| bio1 | 1 |  |  |  |  |  |  |  |  |  |  |  |  |  |  |  |  |  |  |  |  |  |  |
| bio2 | -0.64 | 1 |  |  |  |  |  |  |  |  |  |  |  |  |  |  |  |  |  |  |  |  |  |
| bio3 | -0.11 | 0.30 | 1 |  |  |  |  |  |  |  |  |  |  |  |  |  |  |  |  |  |  |  |  |
| bio4 | -0.33 | 0.38 | -0.71 | 1 |  |  |  |  |  |  |  |  |  |  |  |  |  |  |  |  |  |  |  |
| bio5 | 0.81 | -0.36 | -0.52 | 0.26 | 1 |  |  |  |  |  |  |  |  |  |  |  |  |  |  |  |  |  |  |
| bio6 | 0.91 | -0.74 | 0.13 | -0.6 | 0.52 | 1 |  |  |  |  |  |  |  |  |  |  |  |  |  |  |  |  |  |
| bio7 | -0.45 | 0.60 | -0.54 | 0.96 | 0.12 | -0.7 | 1 |  |  |  |  |  |  |  |  |  |  |  |  |  |  |  |  |
| bio8 | 0.85 | -0.42 | -0.46 | 0.18 | 0.96 | 0.58 | 0.03 | 1 |  |  |  |  |  |  |  |  |  |  |  |  |  |  |  |
| bio9 | 0.89 | -0.61 | 0.22 | -0.67 | 0.51 | 0.97 | -0.74 | 0.55 | 1 |  |  |  |  |  |  |  |  |  |  |  |  |  |  |
| bio10 | 0.88 | -0.49 | -0.484 | 0.13 | 0.98 | 0.64 | -0.01 | 0.98 | 0.61 | 1 |  |  |  |  |  |  |  |  |  |  |  |  |  |
| bio11 | 0.91 | -0.66 | 0.21 | -0.68 | 0.51 | 0.99 | -0.77 | 0.57 | 0.98 | 0.62 | 1 |  |  |  |  |  |  |  |  |  |  |  |  |
| bio12 | 0.64 | -0.80 | 0.02 | -0.53 | 0.28 | 0.75 | -0.67 | 0.35 | 0.67 | 0.42 | 0.72 | 1 |  |  |  |  |  |  |  |  |  |  |  |
| bio13 | 0.57 | -0.73 | 0.03 | -0.45 | 0.23 | 0.66 | -0.59 | 0.34 | 0.56 | 0.38 | 0.63 | 0.95 | 1 |  |  |  |  |  |  |  |  |  |  |
| bio14 | 0.63 | -0.77 | -0.17 | -0.38 | 0.38 | 0.70 | -0.53 | 0.37 | 0.64 | 0.47 | 0.65 | 0.84 | 0.70 | 1 |  |  |  |  |  |  |  |  |  |
| bio15 | -0.53 | 0.58 | 0.23 | 0.21 | -0.41 | -0.54 | 0.32 | -0.36 | -0.52 | -0.45 | -0.50 | -0.47 | -0.26 | -0.64 | 1 |  |  |  |  |  |  |  |  |
| bio16 | 0.58 | -0.74 | 0.07 | -0.50 | 0.22 | 0.68 | -0.63 | 0.32 | 0.60 | 0.36 | 0.66 | 0.97 | 0.99 | 0.72 | -0.31 | 1 |  |  |  |  |  |  |  |
| bio17 | 0.61 | -0.76 | -0.15 | -0.40 | 0.36 | 0.70 | -0.54 | 0.35 | 0.65 | 0.46 | 0.65 | 0.84 | 0.70 | 0.99 | -0.64 | 0.72 | 1 |  |  |  |  |  |  |
| bio18 | 0.55 | -0.70 | 0.09 | -0.47 | 0.19 | 0.64 | -0.61 | 0.31 | 0.55 | 0.33 | 0.63 | 0.93 | 0.98 | 0.63 | -0.25 | 0.98 | 0.64 | 1 |  |  |  |  |  |
| bio19 | 0.59 | -0.73 | -0.12 | -0.40 | 0.33 | 0.67 | -0.54 | 0.31 | 0.64 | 0.42 | 0.63 | 0.83 | 0.68 | 0.97 | -0.62 | 0.70 | 0.98 | 0.61 | 1 |  |  |  |  |
| elevation | -0.70 | 0.45 | 0.67 | -0.38 | -0.91 | -0.40 | -0.21 | -0.92 | -0.33 | -0.92 | -0.37 | -0.37 | -0.38 | -0.40 | 0.35 | -0.35 | -0.38 | -0.34 | -0.34 | 1 |  |  |  |
| slope | -0.02 | 0.01 | 0.03 | -0.02 | -0.03 | -0.01 | -0.01 | -0.04 | -0.01 | -0.03 | -0.01 | 0.01 | 0.01 | -0.01 | -0.01 | 0.01 | -0.01 | 0.01 | -0.01 | 0.04 | 1 |  |  |
| aspect | -0.01 | -0.01 | 0.01 | 0.01 | -0.01 | -0.01 | 0.01 | -0.01 | -0.01 | -0.01 | -0.01 | 0.01 | 0.01 | 0.02 | -0.01 | 0.02 | 0.02 | 0.01 | 0.02 | 0.01 | 0.15 | 1 |  |
| soil | -0.02 | -0.07 | 0.11 | -0.16 | -0.13 | 0.057 | -0.16 | -0.12 | 0.05 | -0.10 | 0.05 | 0.11 | 0.10 | 0.09 | -0.04 | 0.118 | 0.10 | 0.10 | 0.10 | 0.14 | 0.01 | 0.011 | 1 |

Table S3 Distribution records of sour jujube used in Maxent analysis

| location number | Longitude | Latitude | location number | Longitude | Latitude | location number | Longitude | Latitude | location number | Longitude | Latitude | location number | Longitude | Latitude |
| --- | --- | --- | --- | --- | --- | --- | --- | --- | --- | --- | --- | --- | --- | --- |
| 1 | 110.27 | 36.16 | 51 | 108.91 | 38.6 | 101 | 114.86 | 40.83 | 151 | 119.78 | 41.15 | 201 | 118.77 | 36.83 |
| 2 | 109.35 | 35.58 | 52 | 111.25 | 39.83 | 102 | 115.2 | 39.82 | 152 | 119.5 | 41.2 | 202 | 121.41 | 37.52 |
| 3 | 110.56 | 35.38 | 53 | 110.57 | 40.63 | 103 | 117.67 | 41.26 | 153 | 120.01 | 40.3 | 203 | 121.69 | 37.38 |
| 4 | 108.08 | 35.8 | 54 | 120.48 | 44.82 | 104 | 116.1 | 40.79 | 154 | 122.01 | 39.6 | 204 | 120.49 | 37.48 |
| 5 | 110.33 | 37.66 | 55 | 117.23 | 34.66 | 105 | 119.2 | 39.91 | 155 | 122.18 | 40.7 | 205 | 117.73 | 35 |
| 6 | 110.52 | 37.76 | 56 | 112.45 | 37.97 | 106 | 114.19 | 38.9 | 156 | 121.33 | 41.47 | 206 | 117.48 | 35.21 |
| 7 | 110.36 | 38.18 | 57 | 112.48 | 36.08 | 107 | 114.33 | 38.62 | 157 | 122.18 | 40.34 | 207 | 118.06 | 36.32 |
| 8 | 110.32 | 38.31 | 58 | 109.19 | 34.26 | 108 | 114.56 | 40.65 | 158 | 122.53 | 40.65 | 208 | 118.1 | 36.83 |
| 9 | 110.31 | 37.87 | 59 | 109.22 | 34.36 | 109 | 114.05 | 37.91 | 159 | 122.24 | 40.16 | 209 | 117.88 | 36.61 |
| 10 | 110.49 | 38.07 | 60 | 108.99 | 34.01 | 110 | 114.23 | 38.03 | 160 | 122.36 | 39.19 | 210 | 111.27 | 35.22 |
| 11 | 110.44 | 37.11 | 61 | 110.24 | 33.77 | 111 | 114.2 | 38.18 | 161 | 122.91 | 39.66 | 211 | 111.39 | 36.25 |
| 12 | 108.41 | 34.85 | 62 | 110.01 | 34.15 | 112 | 113.96 | 38.05 | 162 | 111.81 | 40.87 | 212 | 113.5 | 35.65 |
| 13 | 113.1 | 33.87 | 63 | 116.2 | 39.99 | 113 | 113.93 | 38.16 | 163 | 110.89 | 39.56 | 213 | 110.76 | 37.46 |
| 14 | 114.28 | 37.22 | 64 | 116.93 | 34.18 | 114 | 114.83 | 39.13 | 164 | 105.94 | 38.76 | 214 | 111.01 | 37.88 |
| 15 | 114.21 | 37.11 | 65 | 118.38 | 29.54 | 115 | 118.6 | 41.03 | 165 | 105.01 | 37.47 | 215 | 111.5 | 39.45 |
| 16 | 114.26 | 37.53 | 66 | 117.06 | 33.89 | 116 | 113.77 | 38.21 | 166 | 106.66 | 38.05 | 216 | 113.92 | 37.95 |
| 17 | 117.5 | 38.99 | 67 | 116.63 | 40.94 | 117 | 114.09 | 38.51 | 167 | 106.03 | 37.75 | 217 | 112.15 | 36.55 |
| 18 | 117.04 | 39.05 | 68 | 116.34 | 39.95 | 118 | 119.83 | 40.04 | 168 | 106.94 | 37.24 | 218 | 110.53 | 34.77 |
| 19 | 116.07 | 40.21 | 69 | 116.33 | 40.2 | 119 | 113.89 | 37.08 | 169 | 105.64 | 37.46 | 219 | 113.73 | 38.77 |
| 20 | 117.1 | 36.5 | 70 | 116.23 | 40.3 | 120 | 114.42 | 40.55 | 170 | 106.07 | 36.43 | 220 | 113.25 | 38.74 |
| 21 | 109.89 | 36.9 | 71 | 115.82 | 39.67 | 121 | 115.23 | 40.28 | 171 | 101.8 | 35.91 | 221 | 113.74 | 37.56 |
| 22 | 110.93 | 34.51 | 72 | 115.6 | 39.84 | 122 | 115.39 | 39.98 | 172 | 102.09 | 35.38 | 222 | 111.49 | 35.42 |
| 23 | 108.15 | 34.66 | 73 | 116.03 | 40.07 | 123 | 113.05 | 34.51 | 173 | 120.67 | 36.26 | 223 | 111.92 | 36.74 |
| 24 | 108.62 | 34.68 | 74 | 115.84 | 40.52 | 124 | 113.73 | 36.15 | 174 | 117 | 35.63 | 224 | 112.45 | 37.71 |
| 25 | 110.32 | 34.58 | 75 | 105.28 | 34.88 | 125 | 110.42 | 34.49 | 175 | 117.15 | 36.23 | 225 | 112.69 | 37.8 |
| 26 | 108.11 | 34.35 | 76 | 108.69 | 36.12 | 126 | 110.72 | 34.33 | 176 | 117.1 | 36.35 | 226 | 111.03 | 37.54 |
| 27 | 109.42 | 36.3 | 77 | 106.11 | 33.73 | 127 | 110.86 | 34.18 | 177 | 117.9 | 34.9 | 227 | 114.16 | 40.41 |
| 28 | 109.46 | 35.81 | 78 | 107.46 | 35.3 | 128 | 111.74 | 33.76 | 178 | 118.9 | 36.7 | 228 | 112.68 | 38.07 |
| 29 | 109.09 | 35.59 | 79 | 105.45 | 33.62 | 129 | 112.51 | 32.99 | 179 | 119.6 | 37 | 229 | 106.59 | 34.11 |
| 30 | 108.98 | 35.01 | 80 | 103.7 | 36.05 | 130 | 114.19 | 31.89 | 180 | 121.23 | 36.85 | 230 | 111.09 | 39.03 |
| 31 | 109.14 | 35.12 | 81 | 107.7 | 35.7 | 131 | 112.28 | 33.14 | 181 | 115.62 | 35.11 | 231 | 109.23 | 36.21 |
| 32 | 109.68 | 35.21 | 82 | 105.73 | 34.55 | 132 | 112.47 | 33.71 | 182 | 117.53 | 36.69 | 232 | 110.22 | 35.59 |
| 33 | 111.91 | 36.84 | 83 | 104.62 | 32.96 | 133 | 112.49 | 34.56 | 183 | 117.26 | 35.77 | 233 | 109.84 | 35.9 |
| 34 | 110.9 | 35.62 | 84 | 104.79 | 32.87 | 134 | 113.18 | 34.66 | 184 | 116.58 | 35.85 | 234 | 106.95 | 33.62 |
| 35 | 109.02 | 32.64 | 85 | 104.53 | 33.02 | 135 | 113.63 | 35.73 | 185 | 117.82 | 36.06 | 235 | 106.17 | 33.35 |
| 36 | 118.12 | 35.1 | 86 | 104.47 | 33.3 | 136 | 111.14 | 32.5 | 186 | 118.63 | 36.2 | 236 | 107.81 | 34.26 |
| 37 | 114.12 | 36.01 | 87 | 104.95 | 33.36 | 137 | 127.28 | 42.34 | 187 | 118.04 | 35.44 | 237 | 110.11 | 37.13 |
| 38 | 112.61 | 34.52 | 88 | 104.54 | 33.43 | 138 | 119.07 | 32.13 | 188 | 118.71 | 34.97 | 238 | 110.36 | 38.73 |
| 39 | 113.52 | 37.98 | 89 | 107.58 | 35.68 | 139 | 120.56 | 31.23 | 189 | 117.93 | 35.54 | 239 | 110.29 | 37.56 |
| 40 | 117.29 | 35.42 | 90 | 113.93 | 36.41 | 140 | 119.15 | 34.55 | 190 | 118.24 | 35.58 | 240 | 107.82 | 34.06 |
| 41 | 116.25 | 40.45 | 91 | 114.36 | 38.05 | 141 | 118.84 | 32.06 | 191 | 120.01 | 36.96 | 241 | 109.06 | 35.22 |
| 42 | 116.67 | 35.09 | 92 | 114.72 | 39.23 | 142 | 119.3 | 34.65 | 192 | 121.07 | 37.2 | 242 | 107.09 | 34.44 |
| 43 | 117.51 | 40.58 | 93 | 114.56 | 37.39 | 143 | 121.26 | 38.82 | 193 | 120.44 | 36.23 | 243 | 109.16 | 35.48 |
| 44 | 110.04 | 34.49 | 94 | 114 | 37.37 | 144 | 119.96 | 40.1 | 194 | 120.09 | 35.99 | 244 | 109.83 | 38.28 |
| 45 | 111.96 | 40.66 | 95 | 114.11 | 37.42 | 145 | 121.54 | 41.49 | 195 | 120.44 | 36.1 | 245 | 108.16 | 34.07 |
| 46 | 108.91 | 40.67 | 96 | 119.39 | 39.77 | 146 | 120.71 | 41.56 | 196 | 119.34 | 35.56 | 246 | 110.13 | 33.77 |
| 47 | 113.78 | 40.47 | 97 | 113.52 | 36.73 | 147 | 121.77 | 39.06 | 197 | 119.12 | 35.46 | 247 | 108.13 | 32.83 |
| 48 | 111.3 | 40.76 | 98 | 114.73 | 39.89 | 148 | 122.44 | 39.4 | 198 | 119.2 | 35.67 | 248 | 108.35 | 34.08 |
| 49 | 118.68 | 41.65 | 99 | 114.97 | 39.95 | 149 | 122.76 | 39.65 | 199 | 117.42 | 35.55 | 249 | 82.96 | 41.72 |
| 50 | 110.02 | 39.83 | 100 | 115.19 | 39.14 | 150 | 119.93 | 40.82 | 200 | 122.36 | 37.3 | 250 | 90.22 | 42.86 |
|  |  |  |  |  |  |  |  |  |  |  |  | 251 | 76.05 | 39.4 |
|  |  |  |  |  |  |  |  |  |  |  |  | 252 | 120.12 | 30.26 |
|  |  |  |  |  |  |  |  |  |  |  |  | 253 | 108.55 | 36.09 |

Table S4 Proportion of membership of sampled populations in Structure analysis

| Genetic cluster | Population | Estimated ancestry | |
| --- | --- | --- | --- |
|  |  | Cluster1 | Cluster 2 |
| Central population | HBQHD | 0.829 | 0.171 |
|  | HBXT | 0.639 | 0.361 |
|  | DTYG | 0.561 | 0.439 |
|  | YCRC | 0.600 | 0.400 |
|  | LYMY | 0.574 | 0.426 |
|  | SDTA | 0.542 | 0.458 |
|  | SXHZ | 0.661 | 0.339 |
|  | SXWN | 0.580 | 0.420 |
|  | HNNY | 0.712 | 0.288 |
|  | HNSMX | 0.621 | 0.379 |
|  | HNLY | 0.712 | 0.288 |
|  | HNZZ | 0.649 | 0.351 |
| Marginal population | HBCD | 0.252 | 0.784 |
|  | CZCZ | 0.480 | 0.520 |
|  | JZTG | 0.342 | 0.658 |
|  | LLXY | 0.320 | 0.680 |
|  | SDLS | 0.258 | 0.742 |
|  | TJWQ | 0.428 | 0.572 |
|  | NXYC | 0.259 | 0.741 |
|  | AHLA | 0.421 | 0.579 |
|  | HBXY | 0.217 | 0.783 |

Table S5 Result of AUC of different periods in MaxEnt modeling

|  | Presence | LGM | MH | LIG | 2050s | | | 2070s | | | mean |
| --- | --- | --- | --- | --- | --- | --- | --- | --- | --- | --- | --- |
|  |  |  |  |  | RCP2.6 | RCP4.5 | RCP8.0 | RCP2.6 | RCP4.5 | RCP8.0 |  |
| /Training data | 0.943 | 0.946 | 0.943 | 0.938 | 0.942 | 0.943 | 0.943 | 0.942 | 0.943 | 0.942 | 0.943 |
| /Test data | 0.932 | 0.937 | 0.933 | 0.928 | 0.931 | 0.933 | 0.932 | 0.932 | 0.933 | 0.931 | 0.932 |

Note: LGM: Last glaciation maximum; MH: middle Holocene; LIG: Last interglacial

Table S6 Contribution of environmental variables in MaxEnt

| Variable | Contribution rate | Accumulated contribution rate |
| --- | --- | --- |
| Bio11 | 35.4 | 35.4 |
| Bio18 | 19.7 | 55.1 |
| Bio7 | 13.3 | 68.4 |
| elevation | 11.9 | 80.3 |
| slope | 8.8 | 89.1 |
| Bio3 | 5.4 | 94.5 |
| Bio19 | 3.6 | 98.1 |
| Bio15 | 1.6 | 99.7 |
| aspect | 0.2 | 99.9 |
| soil | 0.1 | 100 |

Table S7 Distribution of sour jujube in different geological periods

|  | | Present | LGM | MH | LIG | 2050s | | | 2070s | | |
| --- | --- | --- | --- | --- | --- | --- | --- | --- | --- | --- | --- |
|  |  |  |  |  |  | RCP2.6 | RCP4.5 | RCP8.0 | RCP2.6 | RCP4.5 | RCP8.0 |
| Unsuitable area(×10^4^km^2^) | | 853.49 | 867.62 | 851.68 | 790.21 | 855.69 | 859.26 | 864.32 | 859.24 | 863.82 | 865.31 |
| Suitable area (×10^4^km^2^) | | 88.26 | 86.48 | 83.48 | 101.27 | 77.98 | 77.64 | 73.14 | 77.59 | 75.37 | 73.18 |
| Highly suitable area(×10^4^km^2^) | | 20.05 | 7.70 | 26.64 | 70.32 | 28.13 | 24.9 | 24.34 | 24.97 | 22.61 | 23.31 |
| Ratio (%) | HAS | 2.08 | 0.80 | 2.77 | 7.31 | 2.92 | 2.59 | 2.53 | 2.60 | 2.35 | 2.42 |
|  | TSA | 11.26 | 9.79 | 11.45 | 17.84 | 11.03 | 10.66 | 10.14 | 10.66 | 10.19 | 10.03 |

Note: HAS: Higher suitable area; TSA: Total suitable area; LGM: Last glaciation maximum; MH: Middle Holocene; LIG: Last interglacial

Table S8 Nucleotide variation of 21 *Z. jujuba* var. *spinosa* populations in China

| Population | S | H_d_ | π | θ_w_ |
| --- | --- | --- | --- | --- |
| HBCD | 70 | 0.99716 | 0.00733 | 0.00630 |
| HBQHD | 44 | 1 | 0.00572 | 0.00425 |
| HBXT | 55 | 1 | 0.00586 | 0.00499 |
| CZCZ | 63 | 1 | 0.00605 | 0.00648 |
| DTYG | 39 | 1 | 0.00493 | 0.00410 |
| JZTG | 56 | 1 | 0.00640 | 0.00621 |
| LLXY | 55 | 0.98846 | 0.00512 | 0.00597 |
| YCRC | 52 | 0.99858 | 0.00587 | 0.00534 |
| SDLS | 41 | 0.99395 | 0.00462 | 0.00393 |
| LYMY | 66 | 0.99841 | 0.00463 | 0.00702 |
| SDTA | 68 | 1 | 0.00699 | 0.00716 |
| TJWQ | 18 | 0.90196 | 0.00300 | 0.00193 |
| NXYC | 62 | 1 | 0.00611 | 0.00698 |
| SXHZ | 31 | 0.92029 | 0.00313 | 0.00315 |
| SXWN | 42 | 0.99134 | 0.00558 | 0.00464 |
| HNNY | 33 | 0.96842 | 0.00521 | 0.00363 |
| HNSMX | 46 | 0.99638 | 0.00540 | 0.00483 |
| HNLY | 61 | 0.99770 | 0.00614 | 0.00585 |
| HNZZ | 47 | 0.99692 | 0.00587 | 0.00472 |
| AHLA | 33 | 0.99471 | 0.00341 | 0.00331 |
| HBXY | 23 | 0.78077 | 0.00232 | 0.00199 |
